# Youssoufenes A2 and A3, Antibiotic Dimeric Cinnamoyl Lipids from the Δ*dtlA* Mutant of a Marine-Derived *Streptomyces* Strain

**DOI:** 10.3390/md20060394

**Published:** 2022-06-15

**Authors:** Jing Liu, Huayue Li, Zengzhi Liu, Tong Li, Fei Xiao, Wenli Li

**Affiliations:** 1 Key Laboratory of Marine Drugs, Ministry of Education, School of Medicine and Pharmacy, Ocean University of China, Qingdao 266003, China; liujing900908@163.com (J.L.); liuzz1990@outlook.com (Z.L.); oaixlittle@163.com (T.L.); xiaofei3450@ouc.edu.cn (F.X.); 2 Laboratory for Marine Drugs and Bioproducts of Qingdao National Laboratory for Marine Science and Technology, Qingdao 266237, China

**Keywords:** cinnamoyl lipid, benzoic polyene acid, marine-derived *Streptomyces*, multi-drug-resistant, antibiotic

## Abstract

Two new dimeric cinnamoyl lipids (CL) featuring with an unusual dearomatic carbon-bridge, named youssoufenes A2 (**1**) and A3 (**2**), were isolated from the Δ*dtlA* mutant strain of marine-derived *Streptomyces youssoufiensis* OUC6819. Structures of the isolated compounds were elucidated based on extensive MS and NMR spectroscopic analyses, and their absolute configurations were determined by combination of the long-range NOE-based ^1^H-^1^H distance measurements and ECD calculations. Compounds **1** and **2** exhibited moderate growth inhibition against multi-drug-resistant *Enterococcus faecalis* CCARM 5172 with an MIC value of 22.2 μM.

## 1. Introduction

**Figure 1:**
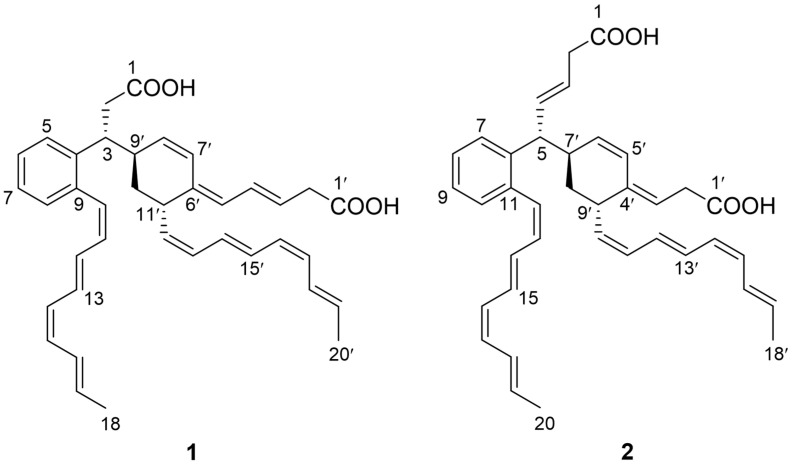
Structures of compounds **1** and **2**.

The *ortho*-substituted cinnamoyl lipids (CL) comprise a small class of secondary metabolites, which are attractive due to their broad bioactive properties, including antibacterial [1], antifungal [2], antitumor [3], antiangiogenic [4] and antituberculosis activities [5,6]. To date, only a small number of CL-containing compounds have been discovered [1,3,7,8,9,10]. Youssoufenes are a series of cryptic compounds which were activated by disruption of aminotransferase family gene *dtlA* in marine-derived *Streptomyces youssoufiensis* OUC6819 [1]. Youssoufenes B1–B4 represent a typical *ortho*-substituted CL skeleton; while youssoufene A1 comprises unique dearomatic carbon-bridged CL dimers [1]. Interestingly, the antibacterial activity of youssoufene A1 against multi-drug-resistant (MDR) *Enterococcus faecalis* was increased 4-fold compared to its monomer [1]. It attracted our interest in the dimeric CL, potentially as a novel drug scaffold. Thus, to discover new dimeric youssoufene analogs, an LC-MS-directed isolation was conducted towards the Δ*dtlA* mutant strain, and two new compounds (**1** and **2**) were obtained (Figure 1). Herein, we describe the isolation, structural elucidation, as well as biological evaluation of these compounds.

## 2. Results and Discussion

The Δ*dtlA* mutant strain, which was constructed in our previous study [1], was cultured for 50 mL, and the culture broth was extracted with EtOAc followed by HPLC-HRESIMS analysis (Figure S1). Except for youssoufene A1, two minor peaks (*m/z* 563 [M + H]^+^]) with similar UV-spectra were observed (Figures S1 and S2), which were proposed to be new dimeric youssoufene analogs. Then, large-scale fermentation (50 L) of the Δ*dtlA* mutant was conducted and afforded compounds **1** and **2**.

**Figure 2:**
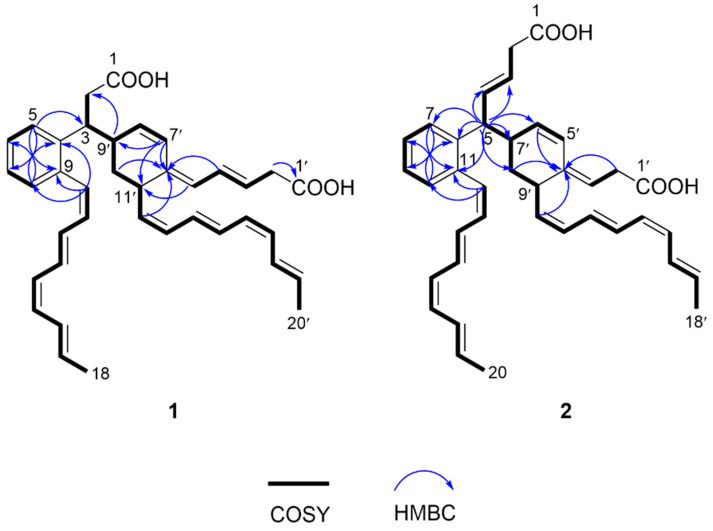
COSY and key HMBC correlations of **1** and **2**.

Compound **1** was isolated as a yellow amorphous solid. The molecular formula of **1** was established as C_38_H_42_O_4_ on the basis of the HRESIMS data ([M + H]^+^ at *m/z* 563.3163, calcd 563.3161), indicating the presence of two additional olefinic carbons compared to youssoufene A1 [1]. The structure of **1** was determined by the NMR data collected in CD_3_OD. In the COSY spectrum of **1**, two methylated olefinic ^1^H spin systems (H-10~H-18 and H-12’~H-20’) (Figure 2) were observed, suggesting the presence of two terminal methyl-octyltetraene chains. The HMBC correlations (Figure 2) from H-5 (*δ*_H_ 7.37) to C-7 (*δ*_C_ 125.6) and C-9 (*δ*_C_ 137.4), from H-8 (*δ*_H_ 7.19) to C-4 (*δ*_C_ 141.2) and C-6 (*δ*_C_ 126.9), and from H-10 (*δ*_H_ 6.83) to C-4, C-8 (*δ*_C_ 130.1) and C-9 revealed the existence of an 4,9-*ortho*-substituted aromatic ring with a methyl-octyltetraene chain at C-9. The COSY correlations between the methylene protons H_2_-2 (*δ*_H_ 2.75, 2.62) and the methine proton H-3 (*δ*_H_ 3.62) (Figure 2), together with the HMBC correlation (Figure 2) from H-5 to C-3 (*δ*_C_ 41.5), confirmed the C-2/C-3 fragment to be connected to the aromatic ring at C-4. The ^1^H spin systems of H_2_-2’ (*δ*_H_ 3.03)~H-5’ (*δ*_H_ 5.70) and H-7’ (*δ*_H_ 6.60)~H-20’ (*δ*_H_ 1.85) in COSY (Figure 2), together with the COSY correlation of H-3/H-9’ (*δ*_H_ 2.68), and the HMBC correlations (Figure 2) from H-7’, H-10’ (*δ*_H_ 1.62, 1.58) and H-12’ (*δ*_H_ 5.24) to the olefinic quaternary carbon C-6’ (*δ*_C_ 135.3), from H-7’ to C-9’ (*δ*_C_ 38.4) and C-11’ (*δ*_C_ 37.1), and from H-9’ to C-2 (*δ*_C_ 38.9), revealed that a 6’,9’,11’-tri-substituted cyclohexene moiety with a methylated octyltetraene chain at C-11’ was connected to C-3. However, unexpectedly, the carboxyl carbons (C-1 and C-1’) were not detected in the NMR spectra of **1** recorded in CD_3_OD. Then, we obtained the NMR data of **1** in DMSO-*d*_6_, from which the presence of carboxyl carbon C-1’ was confirmed by the HMBC correlation from H-2’ (*δ*_H_ 2.80) to C-1’ (*δ*_C_ 174.7) (Figure 2, Table S1), while C-1 was not detected, even in DMSO-*d*_6_. Based on these assigned substructures, together with the HRESIMS data, **1** was determined to be composed of two CL units with terminal carboxyl groups, which formed a dearomatic carbon-bridged dimeric CL skeleton composed of youssoufenes B1 and B3 (or serpentene).

**Figure 3:**
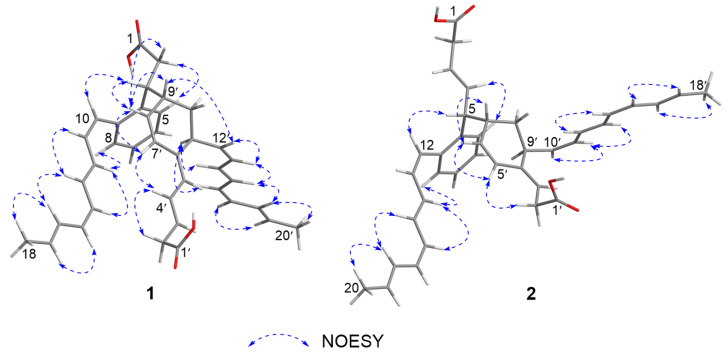
Key NOESY correlations of **1** and **2**.

The NOE correlations of H-10/H-11, H-11/H-13, H-12/H-14, H-13/H-16, H-16/H-18, H-11’/H-14’, H-12’/H-13’, H-13’/H-15’, H-17’/H-19′ and H-18′/H-20′ (Figure 3), together with the vicinal coupling constant values (*^3^J*_H,H_) of *^3^J*_10,11_ (11.3 Hz), *^3^J*_14,15_ (10.8 Hz), *^3^J*_1__2′,1__3′_ (10.3 Hz) and *^3^J*_1__6′,1__7′_ (10.8 Hz) (Table 1) revealed both of the methylated octyltetraene chains in **1** share the same double-bond geometries with youssoufene A1 [1], which were determined as 10-*Z*, 12-*E*, 14-*Z*, 16-*E*, 12′-*Z*, 14′-*E*, 16′-*Z* and 18′-*E*, respectively. The geometries of 3′-*E*, 5′-*E* and 7′-*Z* were determined by combination of the NOEs of H-4′/H-7′, H-5′/H-11′ and H-7′/H-8′ (Figure 3), and the *^3^J*_H,H_ values of *^3^J*_3′,__4′_ (14.8 Hz) and *^3^J*_7′,__8′_ (10.2 Hz). Moreover, the NOE correlation of H-9′/H-12′ suggested *anti*-configuration between H-9′ and H-11′, and the NOEs of H-8′/H-9′ and H-8′/H-3 supported *syn*-configuration between H-3 and H-9′.

**Figure 4:**
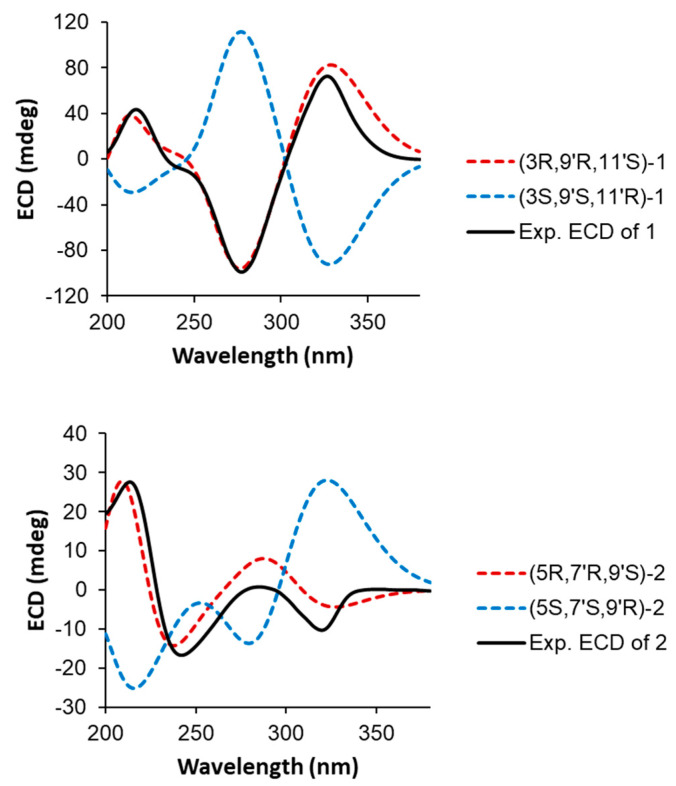
Experimental and calculated ECD spectra of **1** and **2**.

The absolute configurations of C-3, C-9′ and C-11′ in **1** were determined by ECD calculations performed on the CAM-B3LYP/6-31G(d) level of theory with Gaussian 09. The conformers of (3*R*,9′*R*,11′*S*)-**1** or (3*S*,9′*S*,11′*R*)-**1** used in the calculations were selected from the conformers built with SYBYL-X 2.0, in which distances between each atom supported the NOESY data. The calculated ECD curve of (3*R*,9′*R*,11′*S*)-**1** was in good agreement with the experimental ECD data (Figure 4). Thus, compound **1** was finally identified as a new dimeric CL, named youssoufene A2. The ^1^H and ^13^C NMR chemical shift values of **1** are listed in Table 1.

Compound **2** was isolated as a yellow amorphous solid. The molecular formula of **2** was established as C_38_H_42_O_4_ on the basis of the HRESIMS data ([M + H]^+^ at *m/z* 563.3167, calcd 563.3161), indicative of an isomer of youssoufene A2. Compound **2** shares similar NMR data with those of youssoufenes A1 and A2. The difference between **2** and youssoufene A1 was the additional C-3 (*δ*_C_ 125.3)/C-4 (*δ*_C_ 134.1) double bond in **2**, which showed HMBC correlations to H-5 (*δ*_H_ 3.58). Thus, compound **2** was determined to comprise a dimeric CL skeleton with dearomatic youssoufene B1 connected to youssoufene B3/serpentene unit at C-5. By combination of the NOE correlations (Figure 3) and *^3^J*_H,H_ values (Table 1), the double-bond geometries in **2** were determined to be 3-*E*, 12-*Z*, 14-*E*, 16-*Z*, 18-*E*, 3′-*E*, 5′-*Z*, 10′-*Z*, 12′-*E*, 14′-*Z* and 16′-*E*, respectively. The absolute configurations of C-5, C-7′ and C-9′ in **1** were determined by ECD calculations performed on the CAM-B3LYP/6-31G(d) level of theory with Gaussian 09. The calculated ECD curve of (5*R*,7′*R*,9′*S*)-**2** was in good agreement with the experimental ECD data of **2** (Figure 4). Thus, compound **2** was identified as a new dimeric CL, named youssoufene A3. The ^1^H and ^13^C NMR chemical shift values of **2** are listed in Table 1.

In the antibacterial assay, both youssoufenes A2 (**1**) and A3 (**2**) showed growth inhibition against multi-drug-resistant *Enterococcus faecalis* CCARM 5172 with an MIC value of 22.2 μM (Table S2), but not active against *Staphylococcus aureus* CCARM 3090 or *Escherichia coli* CCARM 1009. These results were comparable to that of youssoufene A1, which displayed over 4-fold-increased activity compared to youssoufenes B1–B4 [1]. This result demonstrated that the dimeric CL skeleton endows the youssoufene A-type with notably enhanced antibacterial activities compared to their monomeric B-type structures. While we have demonstrated that monomeric youssoufenes B1-B4 are assembled via an unusual type II polyketide synthetase pathway [11], the diaromatic dimerization to afford youssoufene A-type structures remains unclear.

## 3. Materials and Methods

### 3.1. General Experimental Procedures

Optical rotations were recorded with a JASCO P-1020 digital polarimeter (JASCO, Tokyo, Japan). UV spectra in MeOH were recorded on a PerkinElmer Lambda 35 (PerkinElmer, Waltham, MA, USA). Experimental ECD spectra in MeOH were recorded on a JASCO J-815 spectrometer (JASCO, Tokyo, Japan). IR spectra were measured on a Nicolet NEXUE470 FTIR (Thermo, Waltham, MA, USA). Then, 1D (^1^H and ^13^C) and 2D (COSY, HSQC, HMBC and NOESY) NMR spectra were recorded on a Bruker Avance III 600 spectrometer (Bruker, Billerica, MA, USA). Chemical shifts were reported with reference to the respective solvent peaks and residual solvent peaks (*δ*_H_ 3.31 and *δ*_C_ 49.0 for CD_3_OD; *δ*_H_ 2.50 and *δ*_C_ 39.5 for DMSO-*d*_6_). LC-MS experiments were performed on Agilent 1260 HPLC (Agilent, Santa Clara, CA, USA) system coupled with a Q-TOF Ultima Global GAA076 mass spectrometer (Waters, Milford, MA, USA). Preparative HPLC was performed on a Hitachi Chromaster System (Hitachi, Tokyo, Japan).

### 3.2. LC-MS-Based Production Analyses of the ΔdtlA Mutant Strain of S. youssoufiensis OUC6819

The Δ*dtlA* mutant strain was fermented for 50 mL in the medium (1% soluble starch, 2% glucose, 0.4% corn syrup, 1% yeast extract, 0.3% beef extract, 0.05% MgSO_4_·7H_2_O, 0.05% KH_2_PO_4_, 0.2% CaCO_3_, and 3.3% sea salt, pH = 7.0) at 30 °C, 220 rpm for 7 days. The fermentation supernatant was extracted twice with an equal volume of EtOAc. The resulting EtOAc extract was subjected to HPLC-HRESIMS analysis, eluting with a linear gradient of 20–100% B/A (phase B: 100% ACN + 0.1% HCOOH; phase A: H_2_O + 0.1% HCOOH; flow rate: 0.2 mL/min; wavelength: 300 nm) using an Agilent Eclipse Plus C18 (100 × 2.1 mm, 3.5 μm) (Agilent, Santa Clara, CA, USA) column to trace the youssoufene A analogs.

### 3.3. Fermentation, Extraction and Isolation of the Compounds

A total of 50 L of fermentation culture of the Δ*dtlA* mutant strain were prepared and extracted with EtOAc. The EtOAc extract (8.5 g) was partitioned between 90% MeOH and *n*-hexane to yield two residues. The aqueous MeOH layer (7.1 g) was applied to a reversed-phase (C18) open column (100 × 30 mm) chromatography to give 13 fractions (Fr.1–13) by eluting with gradient from 20% to 100% MeOH. The Fr. 9 (50.4 mg) was further subjected to semipreparative HPLC using a YMC ODS-A column (250 × 10 mm, 5 μm) eluting with 70% ACN to afford compounds **1** (1.1 mg, *t*_R_ 51.9 min) and **2** (1.2 mg, *t*_R_ 55.1 min).

Youssoufene A2 (**1**): yellow amorphous solid; [α]_D_^25^ +5.88 (*c* 0.08, MeOH); UV (MeOH) λ_max_ (log ε) 208 (4.16), 238 (0.65), 280 (4.15), 299 (4.11), 305 (4.13), 314 (4.07), 320 (4.08) nm (Figure S2); ECD (*c* 0.5, MeOH) λ_max_ (Δ*ε*) 216 (+14.83), 277 (−33.69), 326 (+24.77) nm (Figure S3); IR (KBr) *ν*_max_ 3844.6, 3738.0, 3651.0, 3206.4, 1747.2, 1652.6, 1521.1, 1160.1, 1064.2, 980.3, 878.1, 712.6, 608.2, 510.3, 435.5 (Figure S4); ^1^H and ^13^C NMR data, see Table 1; HRESIMS *m/z* 563.3163 [M + H]^+^ (calcd for C_3__8_H_4__3_O_4_, 563.3161).

Youssoufene A3 (**2**): yellow amorphous solid; [α]_D_^25^ −42.38 (*c* 0.08, MeOH); UV (MeOH) λ_max_ (log ε) 207 (4.18), 211 (4.14), 212 (4.15), 227 (4.03), 244 (4.07), 263 (3.98), 292 (4.32), 296 (4.31), 304 (4.45), 312 (4.35), 319 (4.42) nm (Figure S2); ECD (*c* 0.5, MeOH) λ_max_ (Δ*ε*) 213 (+9.39), 242 (−5.64), 285 (0.29), 319 (−3.47), 353 (+0.07) nm (Figure S17); IR (KBr) *ν*_max_ 3852.0, 3742.4, 3612.0, 3134.0, 1745.7, 1648.8, 1520.6, 1174.1, 1063.6, 979.3, 883.5, 739.7, 650.9, 569.9, 463.9 cm^−1^ (Figure S18); ^1^H and ^13^C NMR data, see Table 1; HRESIMS *m/z* 563.3167 [M + H]^+^ (calcd for C_3__8_H_4__3_O_4,_ 563.3161).

### 3.4. Computational Methods

Conformational searches were run by the “Random search” procedure implemented in the SYBYL-X 2.0 program (Certara, Princeton, NJ, USA) using the Molecular Merck force field (MMFF94). Among the generated conformers of each compound, the conformers that well supported the NOESY data were selected and were subjected to geometry optimization with DFT calculations at the TZVP/6-31G(d) level using the Gaussian 09 program (Gaussian, Inc., Pittsburgh, PA, USA). The TD calculations were performed on each optimized conformer using the long-range-corrected hybrid CAM-B3LYP. The number of excited states per molecule was 50. Solvent effects were considered by using the polarizable continuum model (PCM) for MeOH. The ECD spectra were generated by the program GaussView 5.0 (Gaussian, Inc., Pittsburgh, PA, USA).

### 3.5. Antibacterial Activity Assay

The antibacterial activity of compounds **1** and **2** was evaluated using the MIC (minimum inhibitory concentration) assay. The multi-drug-resistant *Enterococcus faecalis* CCARM 5172, *Staphylococcus aureus* CCARM 3090 and *Escherichia coli* CCARM 1009 strains were purchased from Culture Collection of Antimicrobial Resistant Microbes (Seoul Women’s University, Seoul, Korea). The strain was grown overnight at 37 °C in LB medium and diluted with LB broth to 10^6^ cfu/mL. Then, 10 μL of the compound solutions with different concentrations in MeOH were dispensed into 190 μL of the cell suspension in the 96-well plates. LB broth was used as a blank. MeOH was used as a negative control; ciprofloxacin and tetracycline were used as positive controls. The bacterial growth was measured after 18 h of incubation at 37 °C on a microplate reader at a wavelength of 600 nm. Each assay was performed in triplicate.

## 4. Conclusions

In summary, two new dimeric cinnamoyl lipids youssoufenes A2 (**1**) and A3 (**2**), which feature with a unique dearomatic carbon-bridge, were isolated from the Δ*dtlA* mutant strain of marine-derived *S. youssoufiensis* OUC6819. Compounds **1** and **2** exhibited growth inhibition against multi-drug-resistant *E. faecalis* CCARM 5172 (MIC = 22.2 μM), which was comparable to the positive controls, and meanwhile, notably higher than its monomeric form. These results indicated that yousoufene A-type could be an interesting new chemical scaffold for the development of next-generation antibacterial drugs.

## Figures and Tables

**Table 1:** ^1^H (600 MHz) and ^13^C (150 MHz) NMR data of **1** and **2** in CD_3_OD *^a^*.

1			2		
Position	*δ*_C_, type	*δ*_H_ (*J* in Hz)	Position	*δ*_C_, type	*δ*_H_ (*J* in Hz)
1	*^b^*	-	1	*^b^*	-
2	38.9, CH_2_	2.75, 2.62, m	2	*^b^*, CH_2_	2.95, m
3	41.5, CH	3.62, m	3	125.3, CH	5.66, m
4	141.2, C	-	4	134.1, CH	5.74, m
5	126.9, CH	7.37, d (7.8)	5	49.6, CH	3.58, m
6	126.9, CH	7.27, t (7.5)	6	141.4, C	-
7	125.6, CH	7.22, t (7.2)	7	127.1, CH	7.39, d (7.6)
8	130.1, CH	7.19, d (7.4)	8	127.2, CH	7.29, td (7.0, 0.9)
9	137.4, C	-	9	125.3, CH	7.21, m
10	129.9, CH	6.83, d (11.3)	10	129.9, CH	7.19, m
11	130.8, CH	6.43, m	11	136.5, C	-
12	129.3, CH	6.42, m	12	129.3, CH	6.60, m
13	129.9, CH	6.83, t (13.0)	13	130.9, CH	6.46, m
14	127.1, CH	5.84, t (10.8)	14	129.1, CH	6.33, m
15	129.6, CH	5.98, t (10.8)	15	129.8, CH	6.85, dd (14.2, 11.9)
16	127.2, CH	6.64, m	16	127.0, CH	5.83, m
17	130.3, CH	5.79, m	17	129.5, CH	5.97, m
18	17.3, CH_3_	1.85, d (6.7)	18	127.2, CH	6.63, m
1′	*^a^*	-	19	130.2, CH	5.78, m
2′	40.5, CH_2_	3.03, m	20	17.3, CH_3_	1.84, d (6.7)
3′	127.7, CH	5.73, m	1′	*^a^*	-
4′	127.4, CH	6.48, dd (14.8, 11.9)	2′	35.1, CH_2_	3.03, m
5′	125.3, CH	5.70, m	3′	120.1, CH	5.41, m
6′	135.3, C	-	4′	136.5, C	-
7′	123.9, CH	6.60, d (10.2)	5′	123.1, CH	6.33, m
8′	131.2, CH	5.71, t (10.9)	6′	131.7, CH	5.49, d (10.6, 9.1)
9′	38.4, CH	2.68, m	7′	38.3, CH	2.71, m
10′	32.7, CH_2_	1.62, m 1.58, m	8′	33.6, CH_2_	1.74, m 1.63, m
11′	37.1 CH	3.19, m	9′	37.5, CH	3.48, m
12′	133.7, CH	5.24, t (10.3)	10′	134.1, CH	5.41, m
13′	128.4, CH	6.05, t (10.3)	11′	128.0, CH	6.08, t (11.0)
14′	128.1, CH	6.30, dd (14.1, 12.0)	12′	127.9, CH	6.49, m
15′	128.5, CH	6.70, dd (14.6, 10.8)	13′	128.4, CH	6.71, m
16′	127.1, CH	5.96, m	14′	127.2, CH	5.97, m
17′	129.7, CH	5.98, t (10.8)	15′	130.2, CH	5.97, m
18′	127.2, CH	6.64, m	16′	127.2, CH	6.63, m
19′	130.5, CH	5.79, m	17′	130.7, CH	5.78, m
20′	17.3, CH_3_	1.85, d (6.7)	18′	17.3, CH_3_	1.84, d (6.7)

*^a^* ^13^C chemical shifts were obtained by combination of ^13^C NMR, HSQC and HMBC analysis*^; b^* not detected.

## Data Availability

The data presented in this study are available on request from the corresponding author.

## Supplementary Materials

The following supporting information can be downloaded at: https://www.mdpi.com/article/10.3390/md20060394/s1, Figures S1–S25: the spectroscopic data (HPLC-HRESIMS, NMR, UV and ECD) of compounds **1** and **2**; Table S1: NMR data of compound **1** in DMSO-*d*_6_; Table S2: antibacterial activity of compounds **1** and **2**; Table S3: bacteria and plasmids; Tables S4–S7: the coordinates of the selected conformers for ECD calculations. Refs. [12,13] are listed in Table S3.
