# Supplementary material for: Youssoufenes A2 and A3, Antibiotic Dimeric Cinnamoyl Lipids from the ΔdtlA Mutant of a Marine-Derived Streptomyces Strain
Source: Mar Drugs. 2022 Jun 15;20(6):394. doi: 10.3390/md20060394 (PMC9230751; doi:10.3390/md20060394)
Supplement: Supplementary file 1 [file marinedrugs-20-00394-s001.zip › marinedrugs-1670907-supplementary.pdf]

Supporting Information

Youssoufenes A2 and A3, Antibiotic Dimeric Cinnamoyl Lipids from the *ΔdtlA* Mutant of a Marine-Derived *Streptomyces* Strain

Jing Liu[1,†], Huayue Li[1,2,*,†], Zengzhi Liu[1], Tong Li[1], Zirong Deng[1], Fei Xiao[1], Wenli Li[1,2,*]

[1]Key Laboratory of Marine Drugs, Ministry of Education, School of Medicine and Pharmacy, Ocean University of China, Qingdao 266003, China

[2]Laboratory for Marine Drugs and Bioproducts of Qingdao National Laboratory for Marine Science and Technology, Qingdao 266237, China

Table of contents

Figure S1. HPLC-HRESIMS of the culture extract of the *ΔdtlA* mutant strain of marine-derived *S. youssoufiensis* OUC6819
Figure S2. UV spectrum of compounds **1** and **2**
Figure S3. ECD spectrum of compound **1**
Figure S4. IR (KBr) spectrum of compound **1**
Figure S5. HRESIMS spectrum of compound **1**
Figure S6. ^{1}H NMR spectrum of compound **1** in CD_3OD (600 MHz)
Figure S7. ^{13}C NMR spectrum of compound **1** in CD_3OD (150 MHz)
Figure S8. COSY spectrum of compound **1** in CD_3OD (600 MHz)
Figure S9. HSQC spectrum of compound **1** in CD_3OD (600 MHz)
Figure S10. HMBC spectrum of compound **1** in CD_3OD (600 MHz)
Figure S11. NOESY spectrum of compound **1** in CD_3OD (600 MHz)
Figure S12. ^{1}H NMR spectrum of compound **1** in DMSO-d_6 (600 MHz)
Figure S13. COSY spectrum of compound **1** in DMSO-d_6 (600 MHz)
Figure S14. HSQC spectrum of compound **1** in DMSO-d_6 (600 MHz)
Figure S15. HMBC spectrum of compound **1** in DMSO-d_6 600 MHz)
Figure S16. NOESY spectrum of compound **1** in DMSO-d_6 (600 MHz)
Figure S17. ECD spectrum of compound **2**
Figure S18. IR (KBr) spectrum of compound **2**
Figure S19. HRESIMS spectrum of compound **2**
Figure S20. ^{1}H NMR spectrum of compound **2** in CD_3OD (600 MHz)
Figure S21. ^{13}C NMR spectrum of compound **2** in CD_3OD (150 MHz)
Figure S22. COSY spectrum of compound **2** in CD_3OD (600 MHz)
Figure S23. HSQC spectrum of compound **2** in CD_3OD (600 MHz)
Figure S24. HMBC spectrum of compound **2** in CD_3OD (600 MHz)
Figure S25. NOESY spectrum of compound **2** in CD_3OD (600 MHz)
Table S1. ^{1}H (600 MHz) and ^{13}C (150 MHz) NMR data of compound **1** in DMSO-d_6
Table S2. Antibacterial activity of compounds **1** and **2** against multi-drug resistant *Enterococcus faecalis* CCARM 5172
Table S3. Bacteria and plasmids used in this study
Table S4. The computational method and coordinates of (3*R*,9'*R*,11'*S*)-**1** for ECD calculation
Table S5. The computational method and coordinates of (3*S*,9'*S*,11'*R*)-**1** for ECD calculation
Table S6. The computational method and coordinates of (5*R*,7'*R*,9'*S*)-**2** for ECD calculation
Table S7. The computational method and coordinates of (5*S*,7'*S*,9'*R*)-**2** for ECD calculation
References

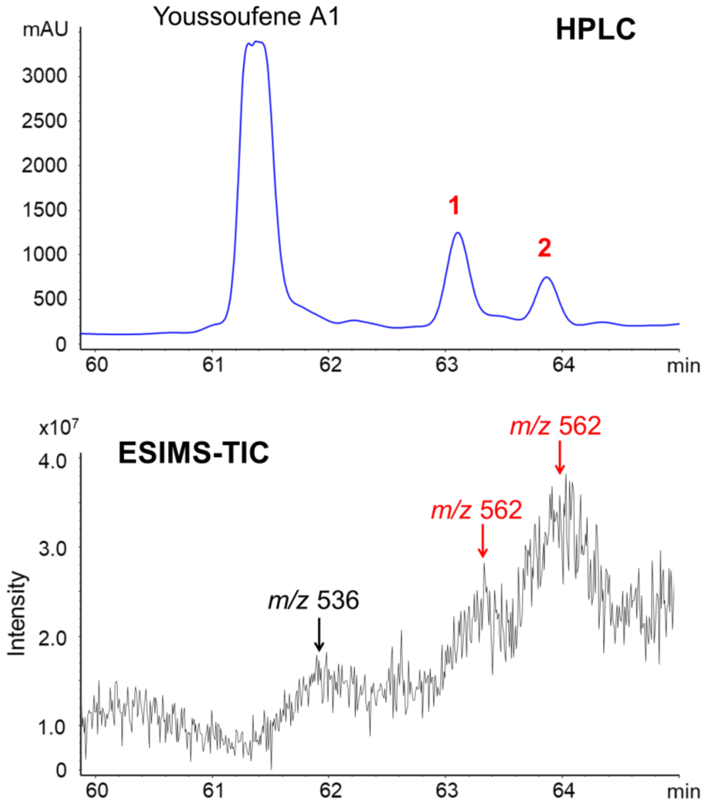

Figure S1. HPLC-HRESIMS of the culture extract of the *ΔdtlA* mutant strain of marine-derived *S. youssoufiensis* OUC6819. HPLC conditions: Agilent Eclipse Plus C18 (100 × 2.1 mm, 3.5 μm); 20-100% gradient ACN/H_2O; wavelength 300 nm.

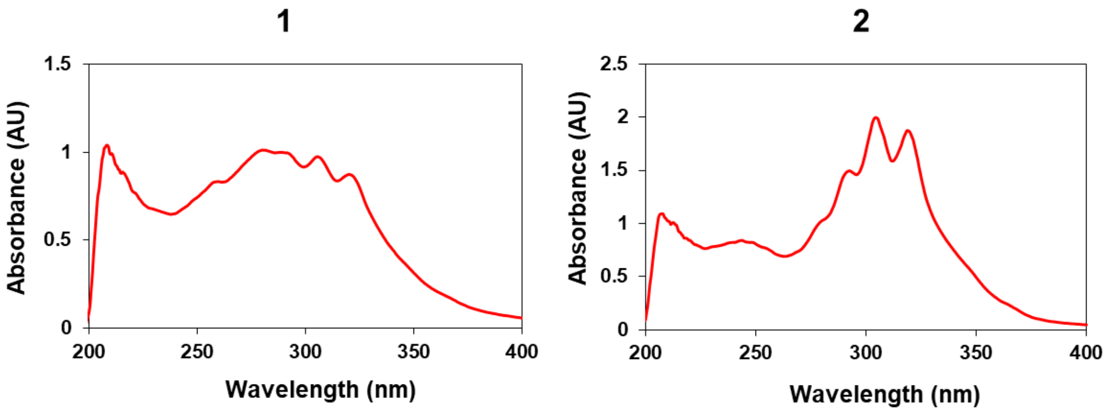

Figure S2. UV spectra of compounds **1** and **2**.

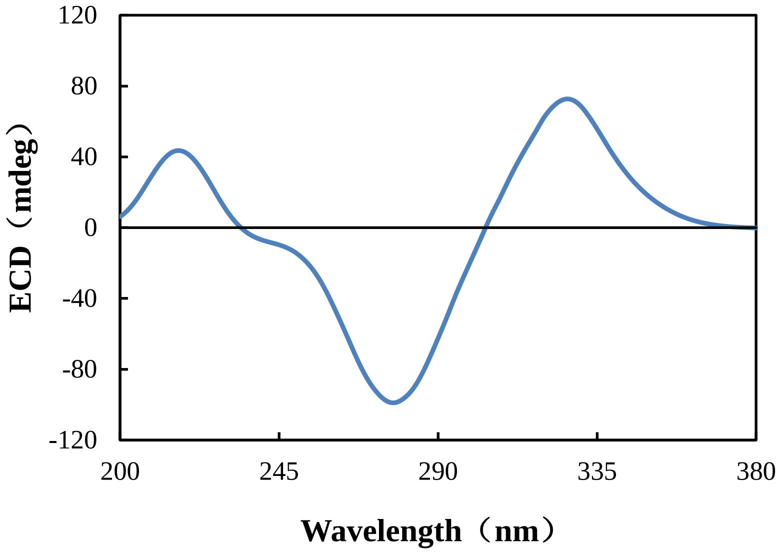

Figure S3. ECD spectrum of compound **1**

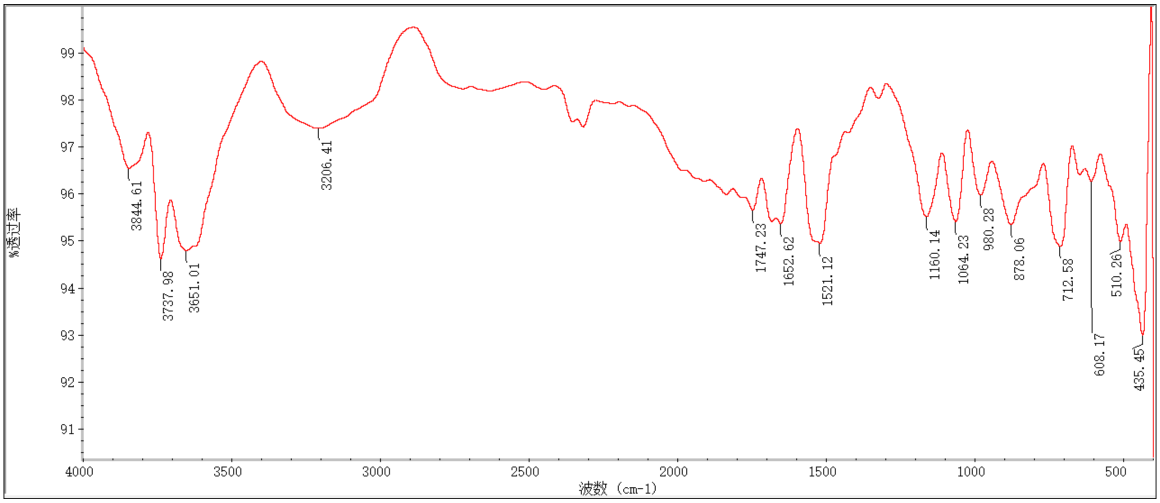

Figure S4. IR (KBr) spectrum of compound **1**

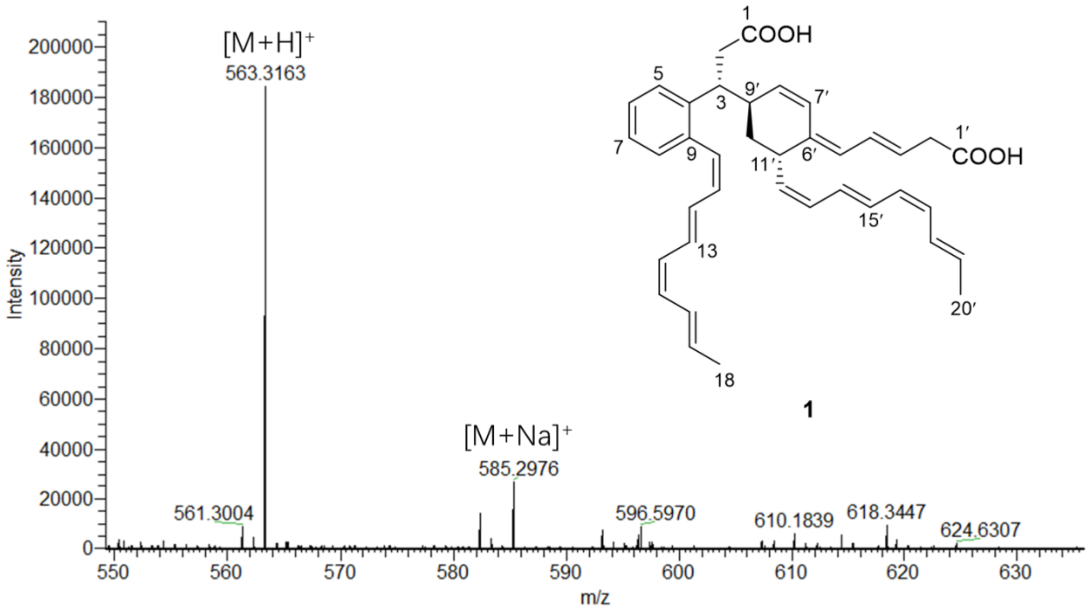

Figure S5. HR-ESIMS spectrum of compound **1**.

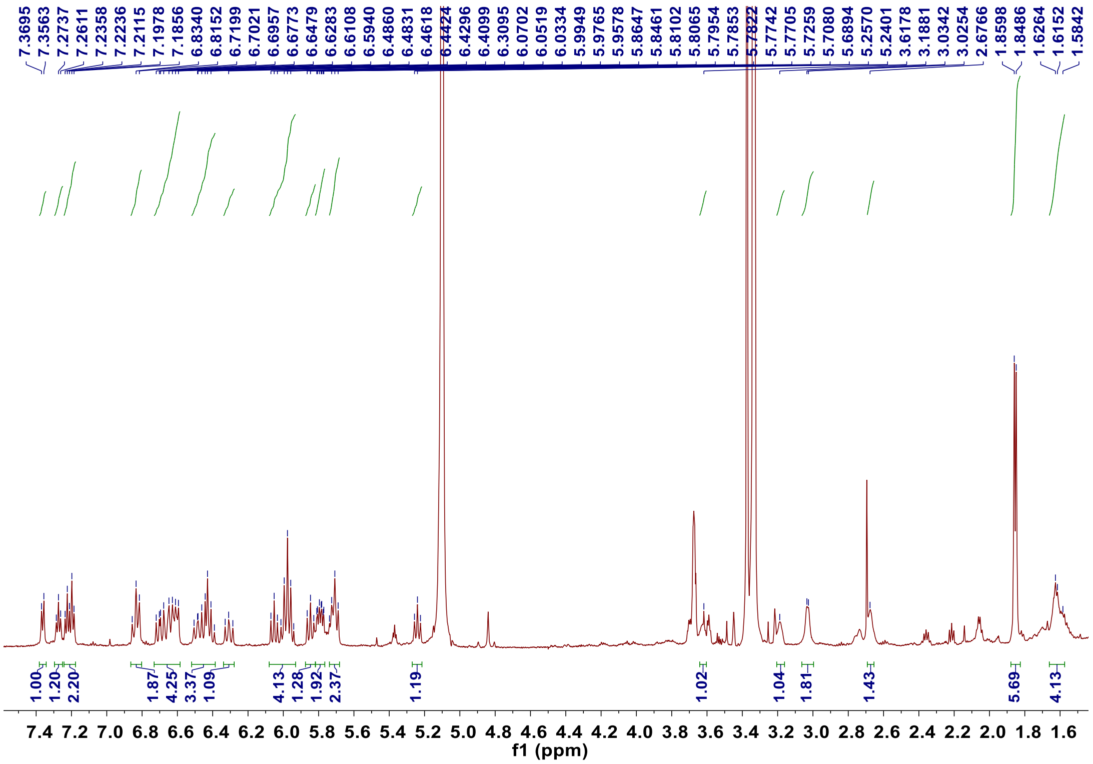

Figure S6. ^{1}H NMR spectrum of compound **1** in CD_3OD (600 MHz).

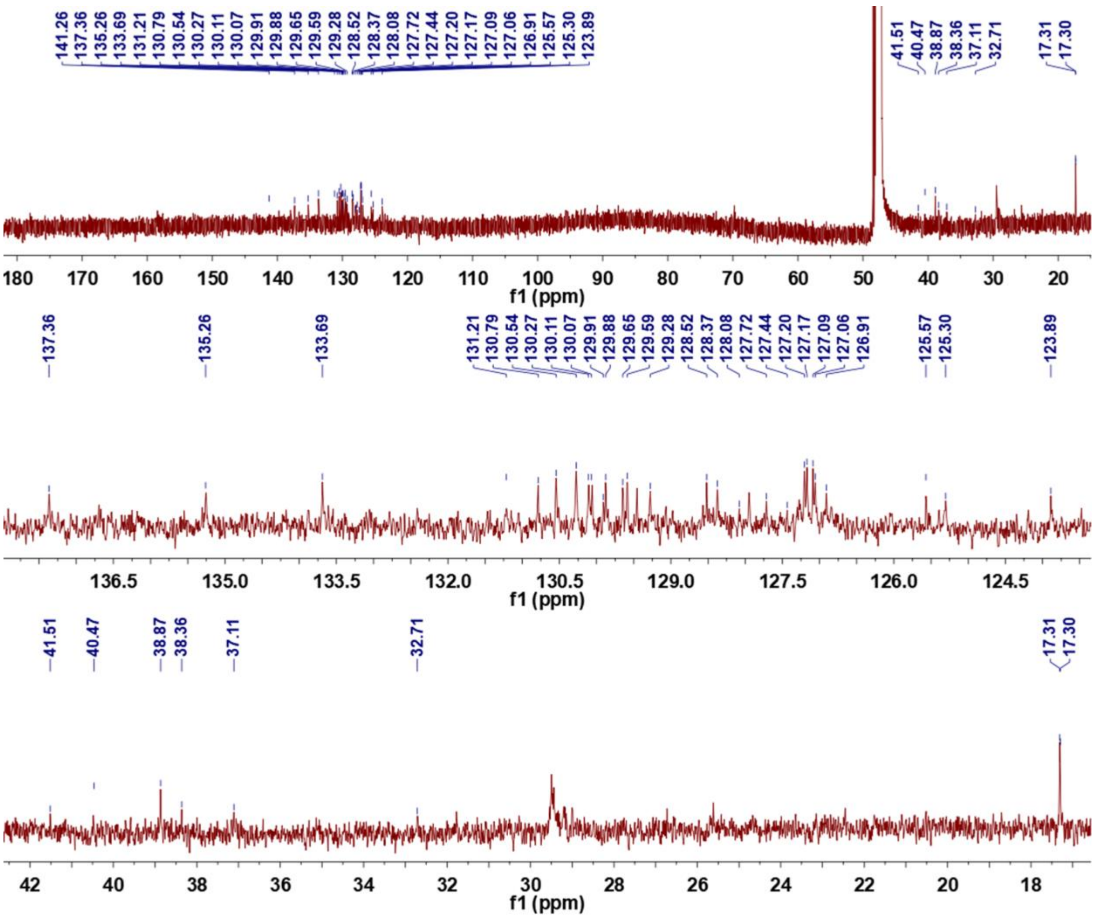

Figure S7. ^{13}C NMR spectrum of compound **1** in CD_3OD (150 MHz).

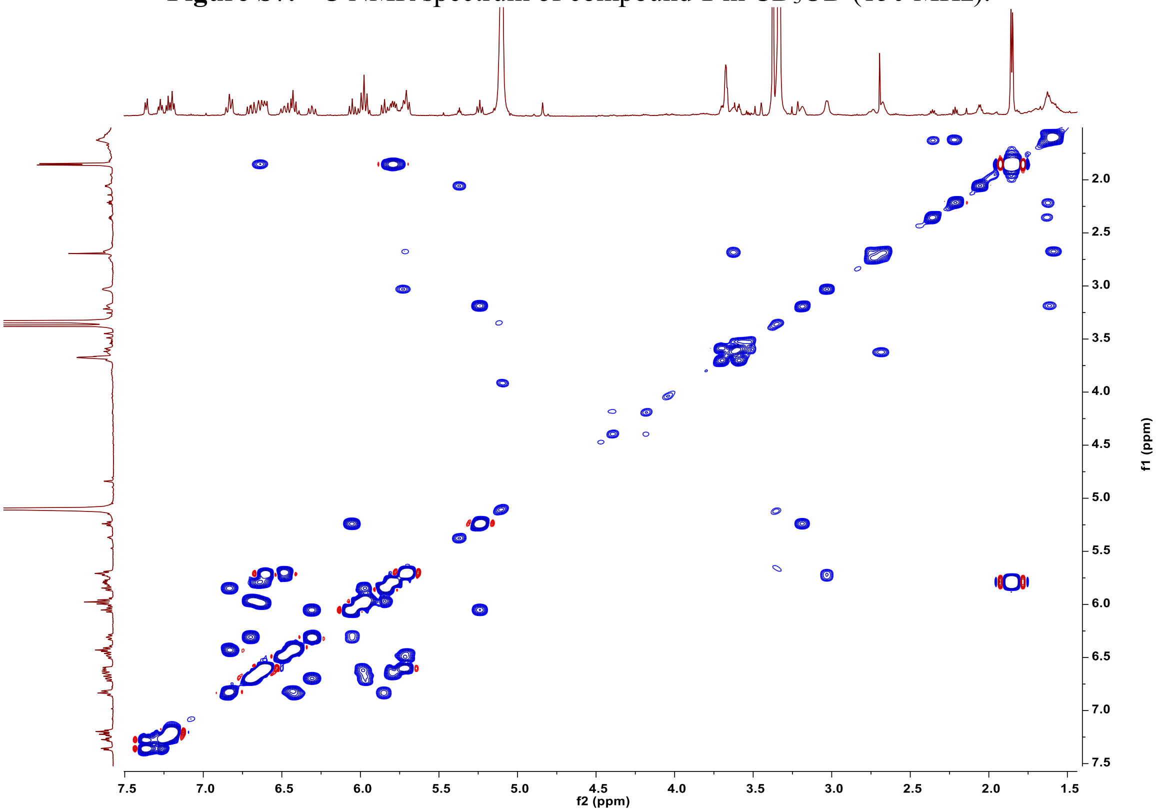

Figure S8. COSY spectrum of compound **1** in CD_3OD (600 MHz).

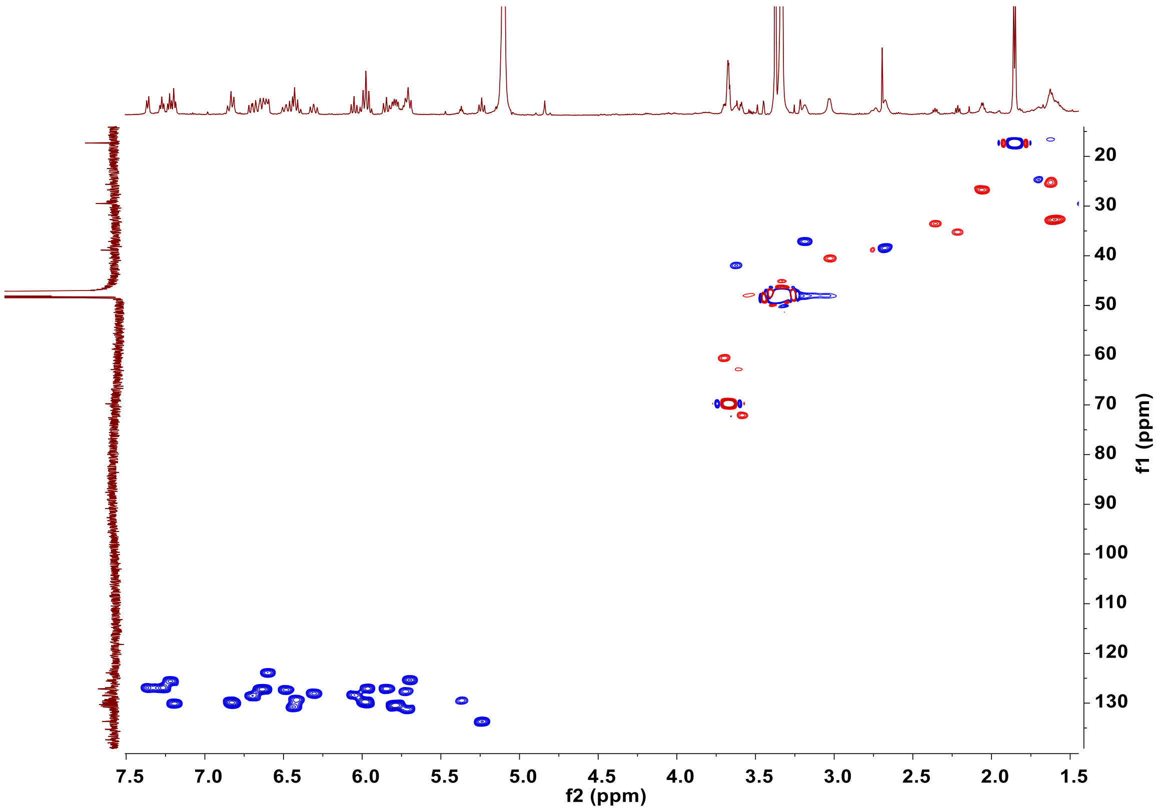

Figure S9. HSQC spectrum of compound **1** in CD_3OD (600 MHz).

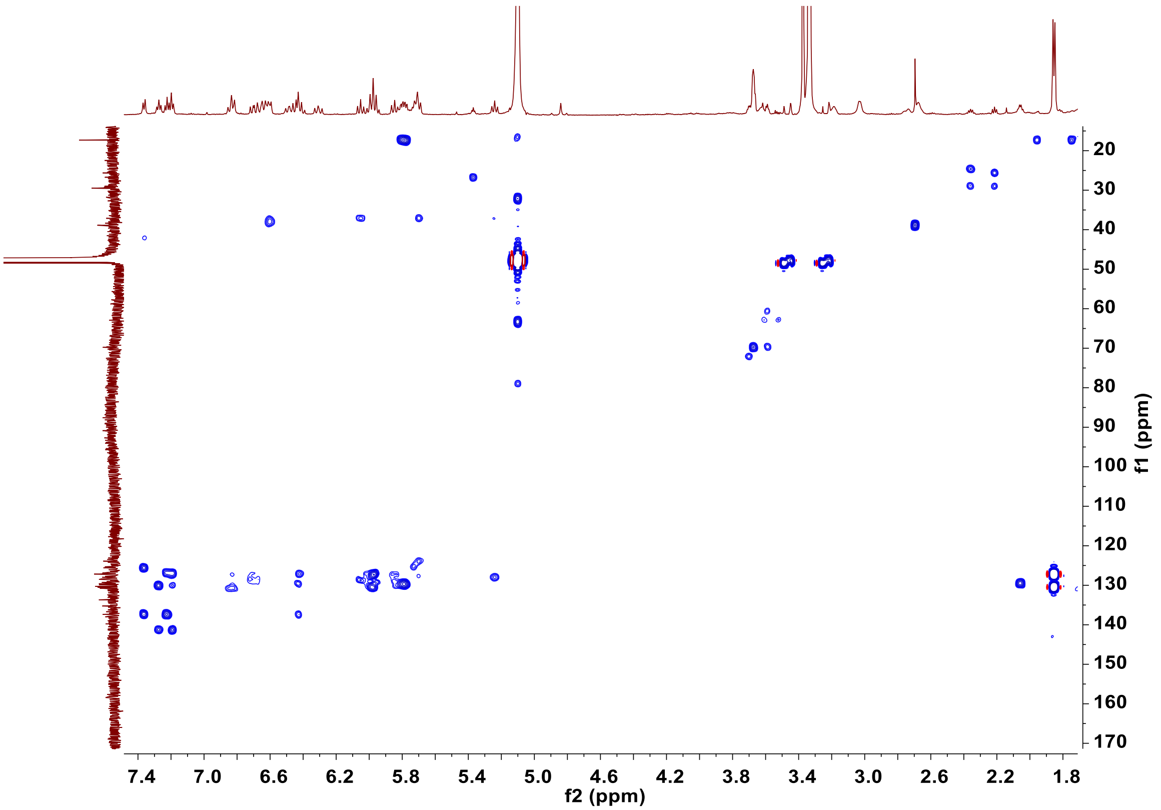

Figure S10. HMBC spectrum of compound **1** in CD_3OD (600 MHz).

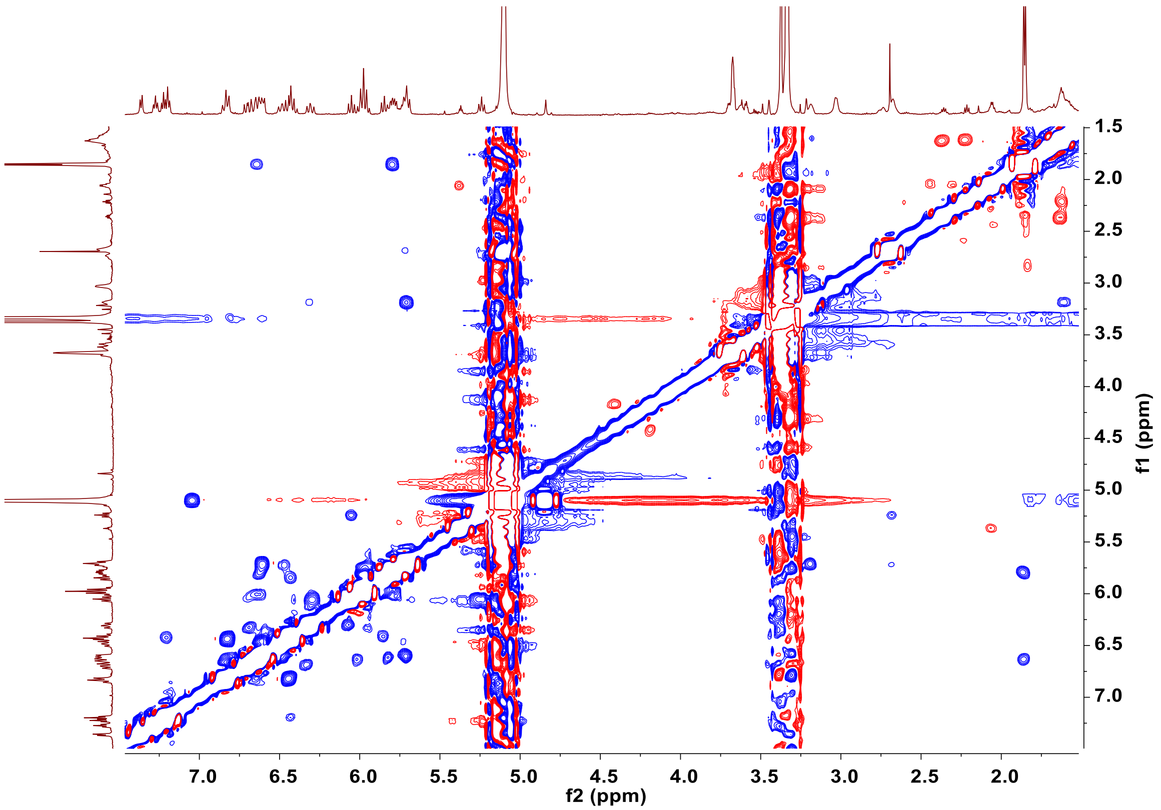

Figure S11. NOESY spectrum of compound **1** in CD_3OD (600 MHz)

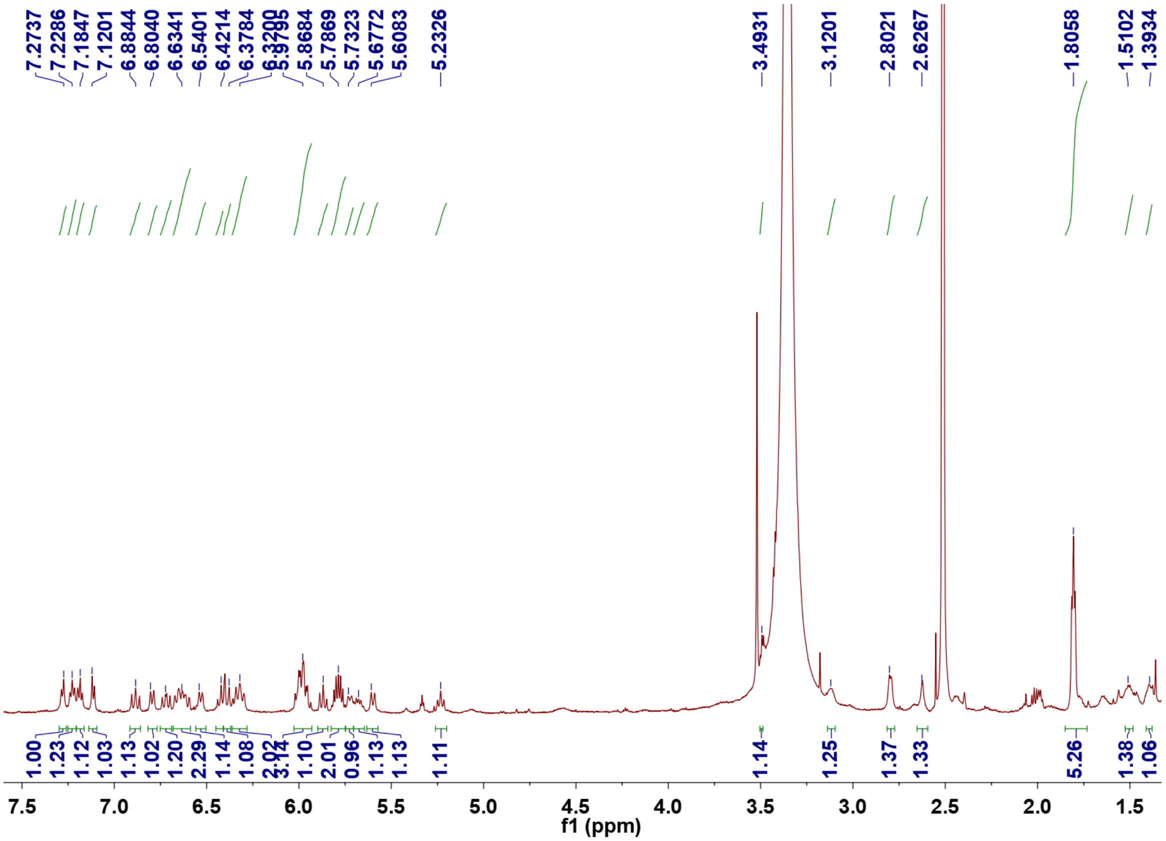

Figure S12. ^{1}H NMR spectrum of compound **1** in DMSO-d_6 (600 MHz).

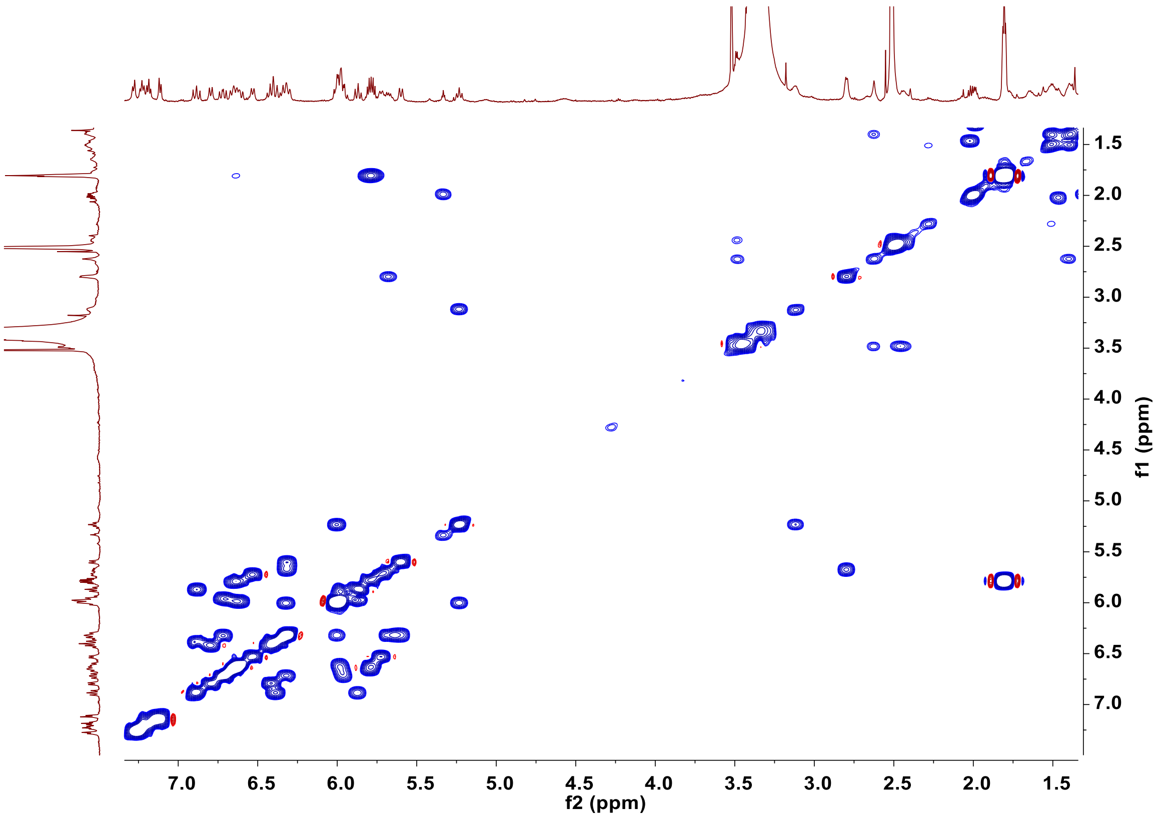

Figure S13. COSY spectrum of compound **1** in DMSO-d_6 (600 MHz).

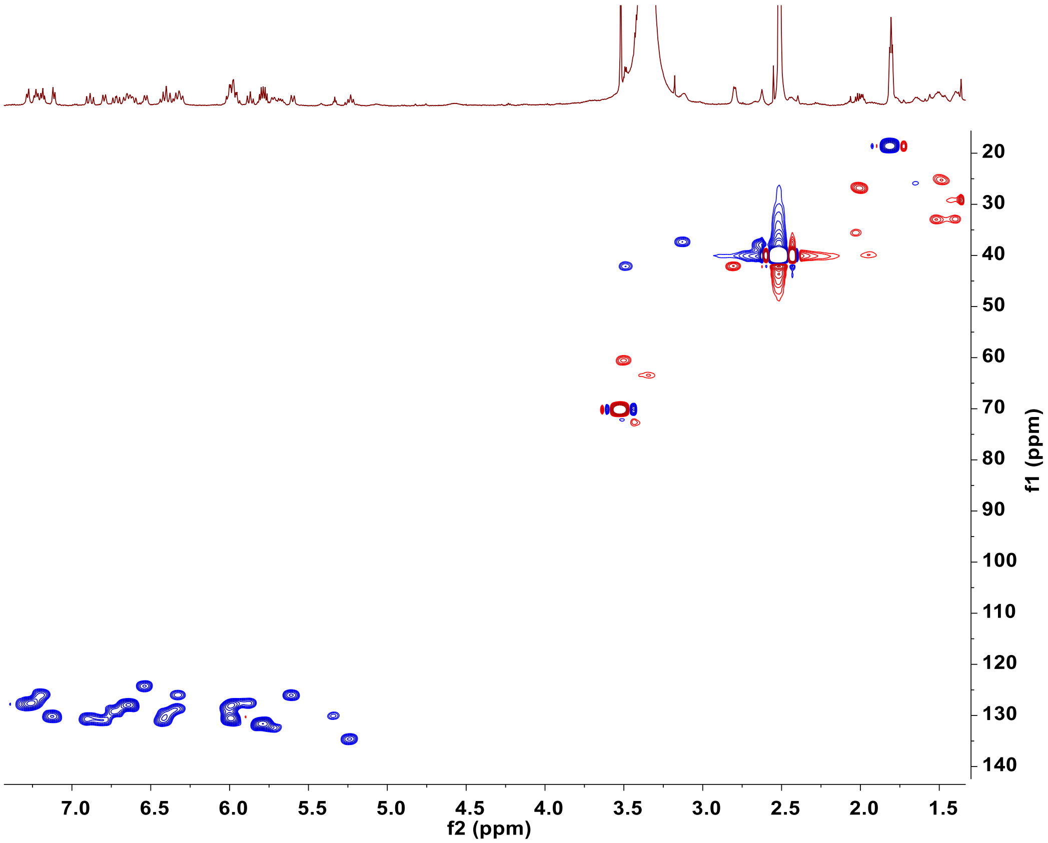

Figure S14. HSQC spectrum of compound **1** in DMSO-d_6 (600 MHz).

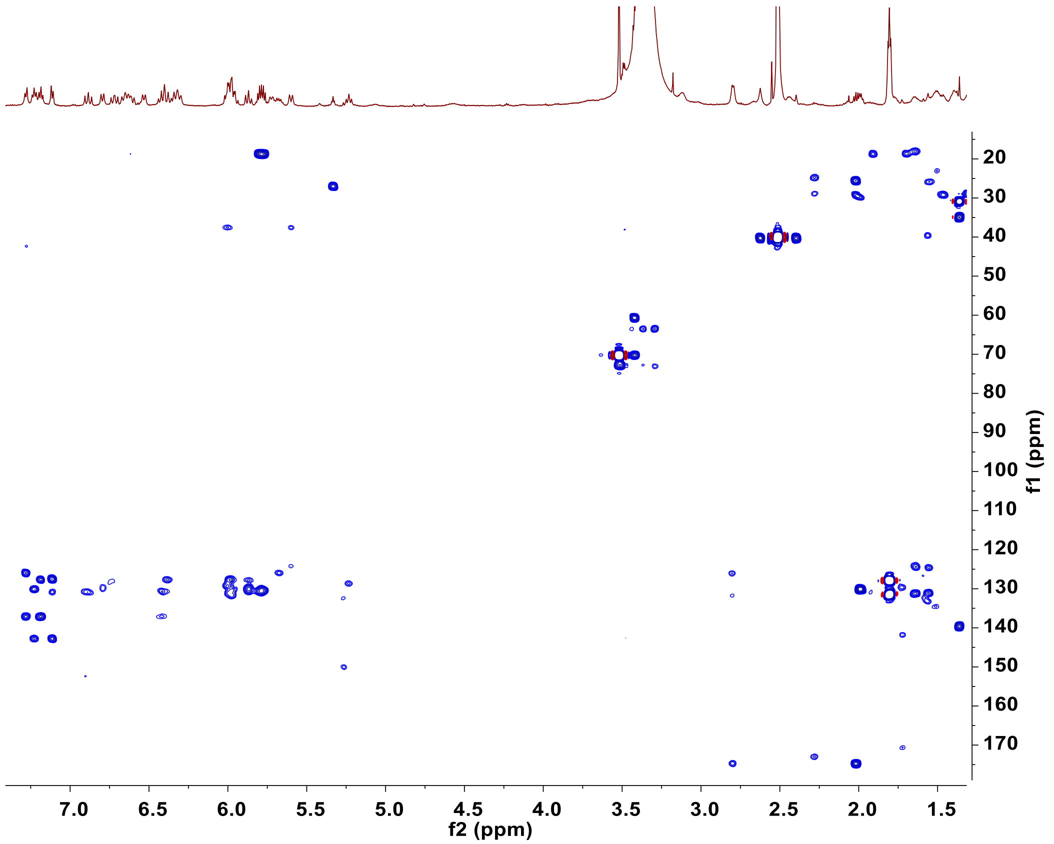

Figure S15. HMBC spectrum of compound **1** in DMSO-d_6 (600 MHz).

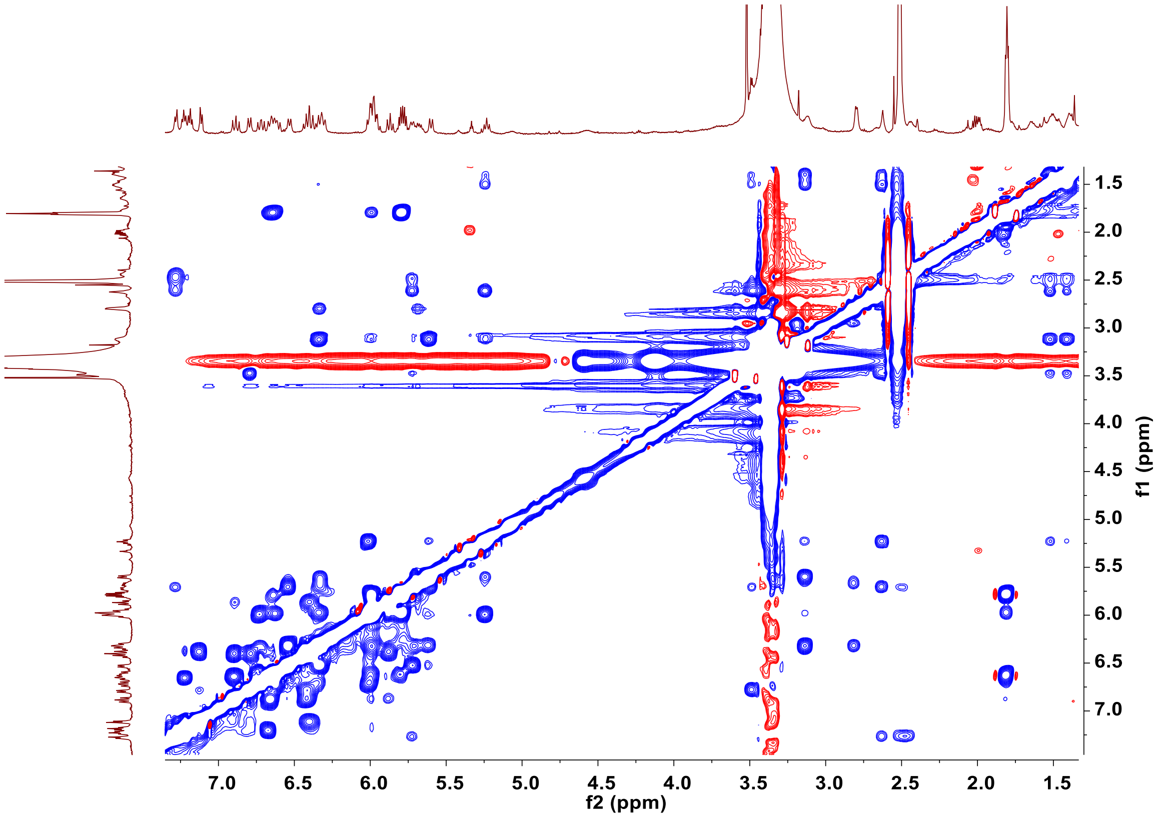

Figure S16. NOESY spectrum of compound **1** in DMSO-d_6 (600 MHz)

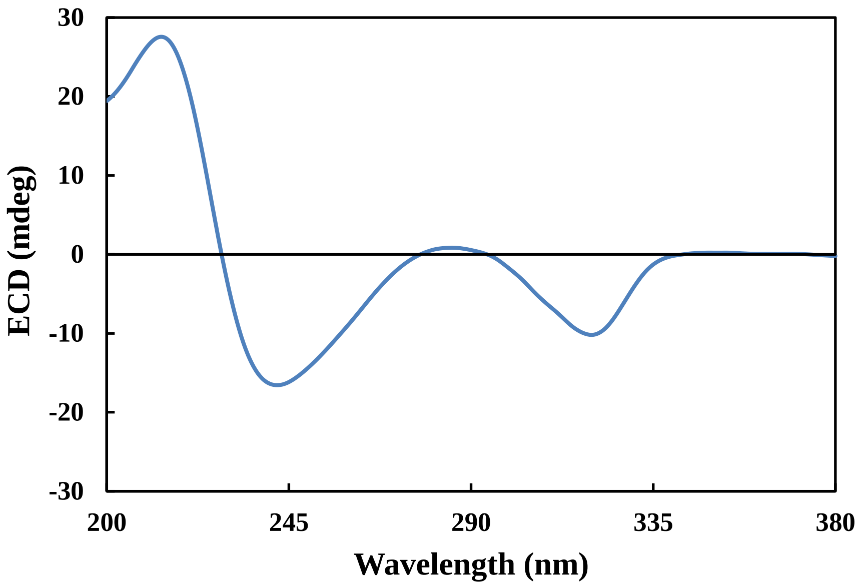

Figure S17. ECD spectrum of compound **2**

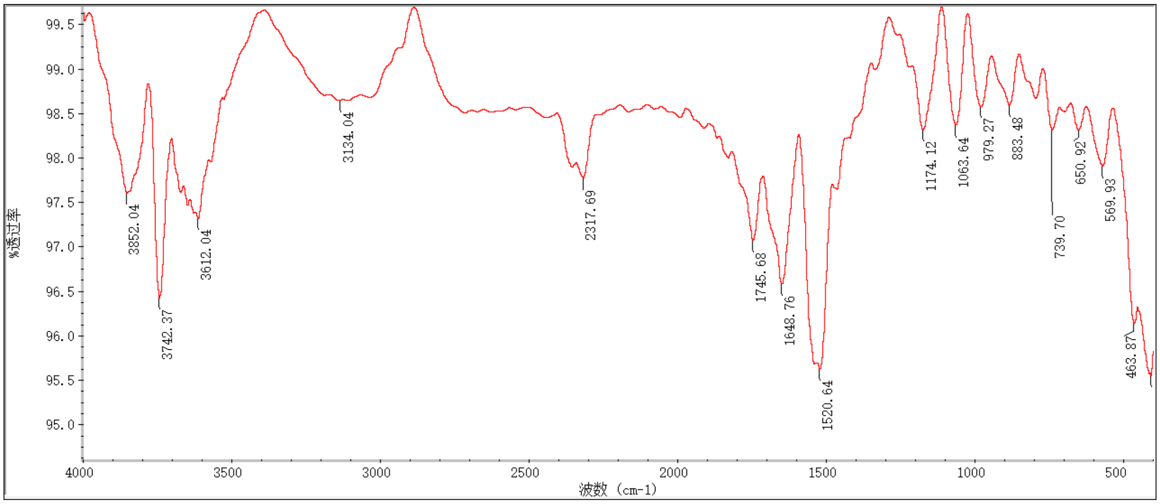

Figure S18. IR (KBr) spectrum of compound **2**

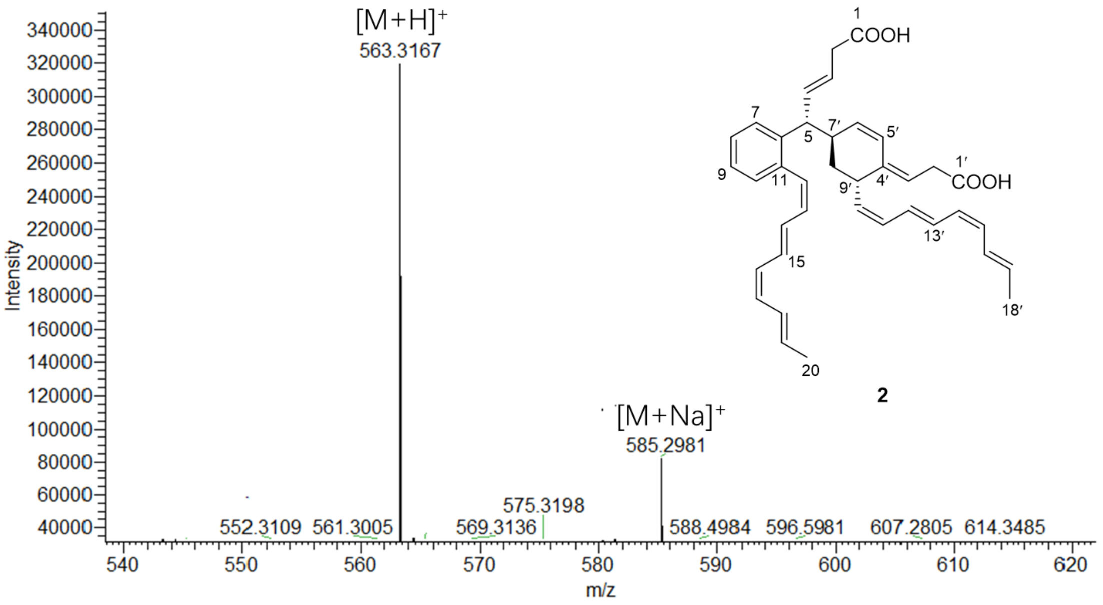

Figure S19. HR-ESIMS spectrum of compound **2**

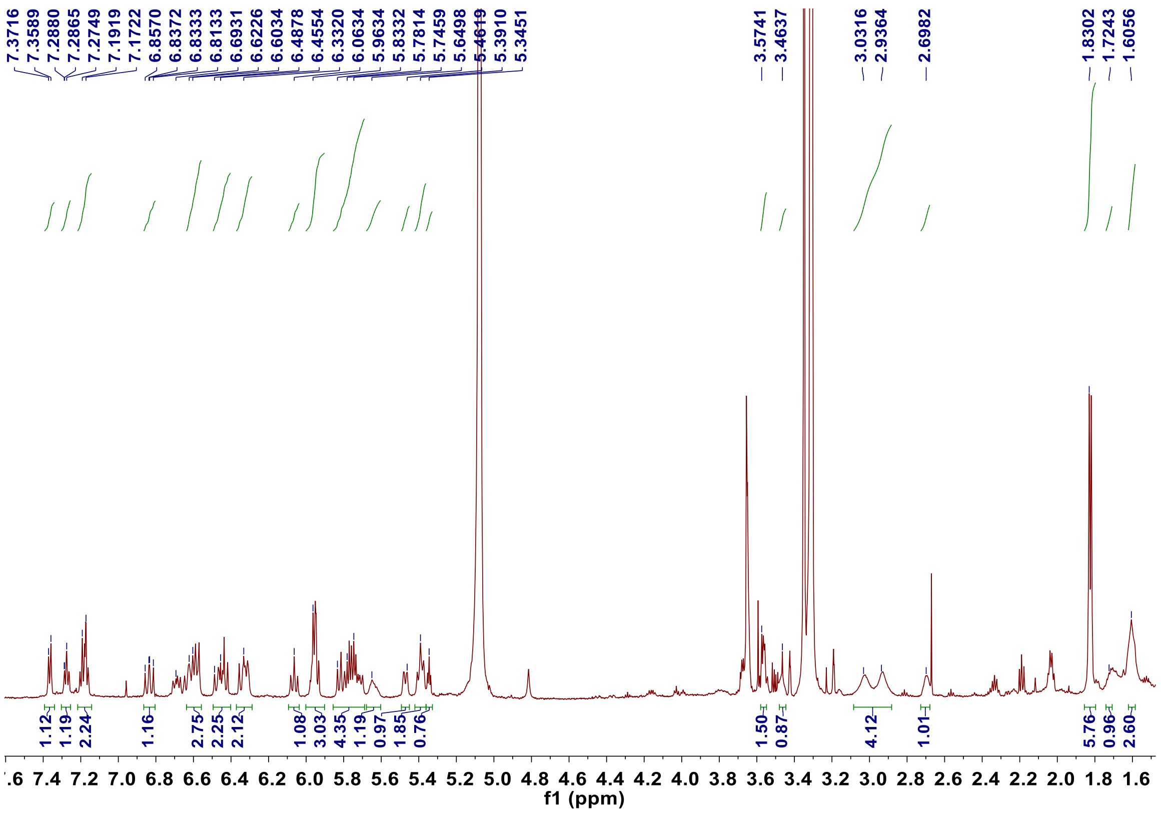

Figure S20. ^{1}H NMR spectrum of compound **2** in CD_3OD (600 MHz)

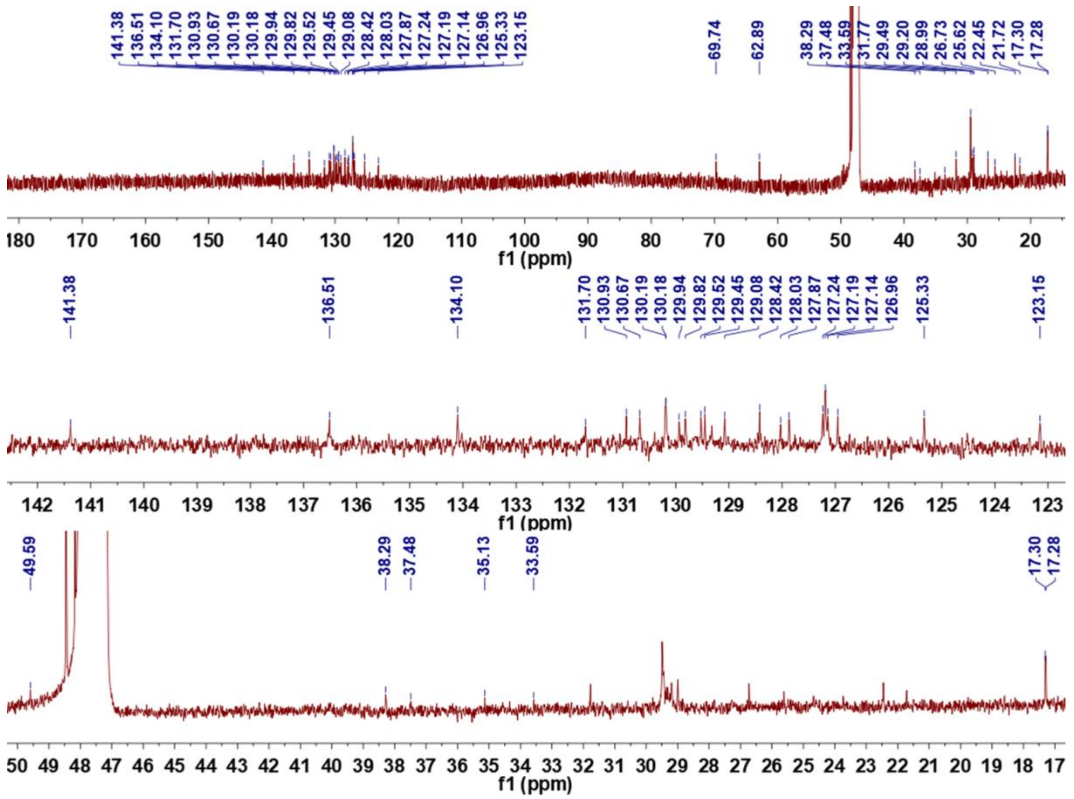

Figure S21. ^{13}C NMR spectrum of compound **2** in CD_3OD (150 MHz)

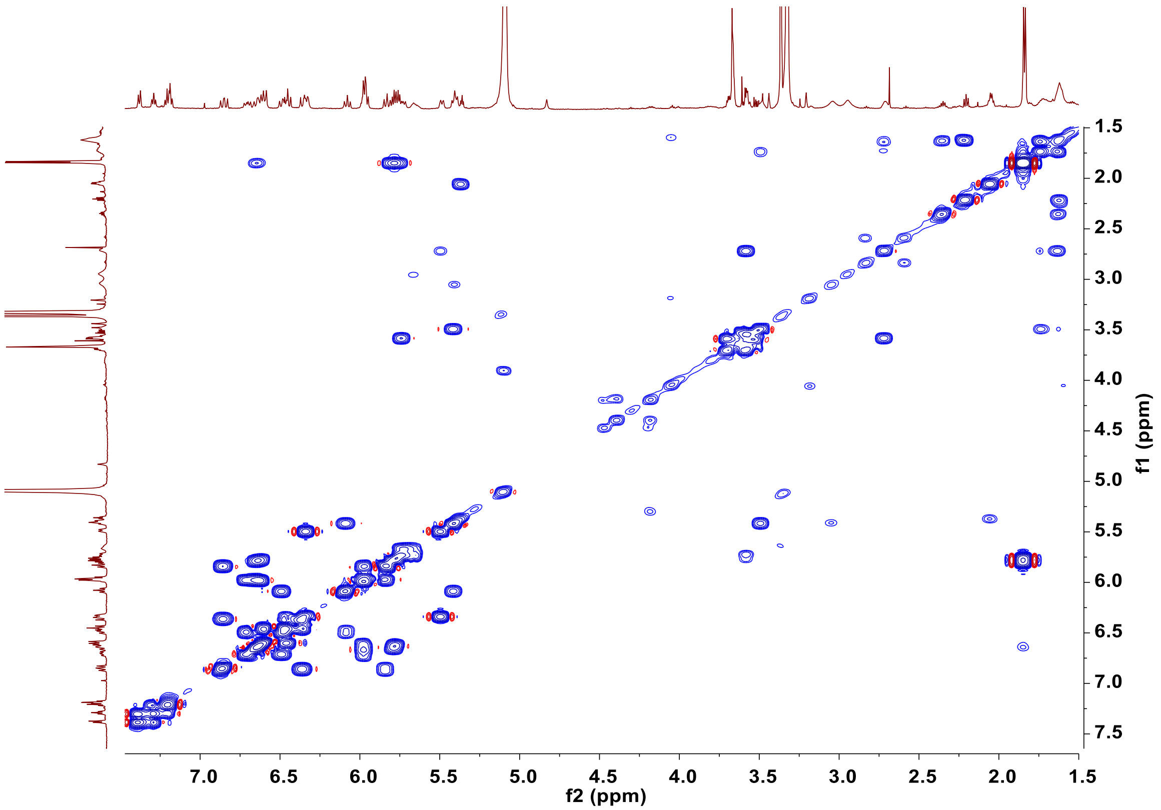

Figure S22. COSY spectrum of compound **2** in CD_3OD (600 MHz)

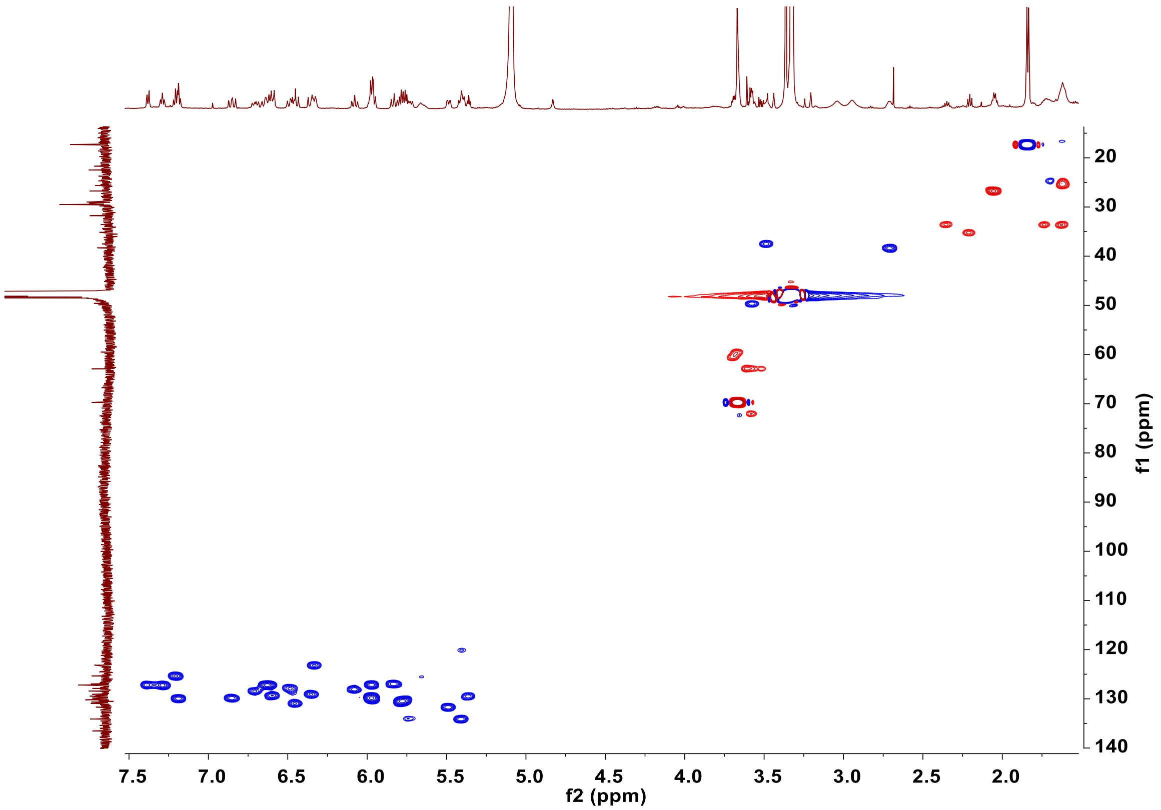

Figure S23. HSQC spectrum of compound **2** in CD_3OD (600 MHz)

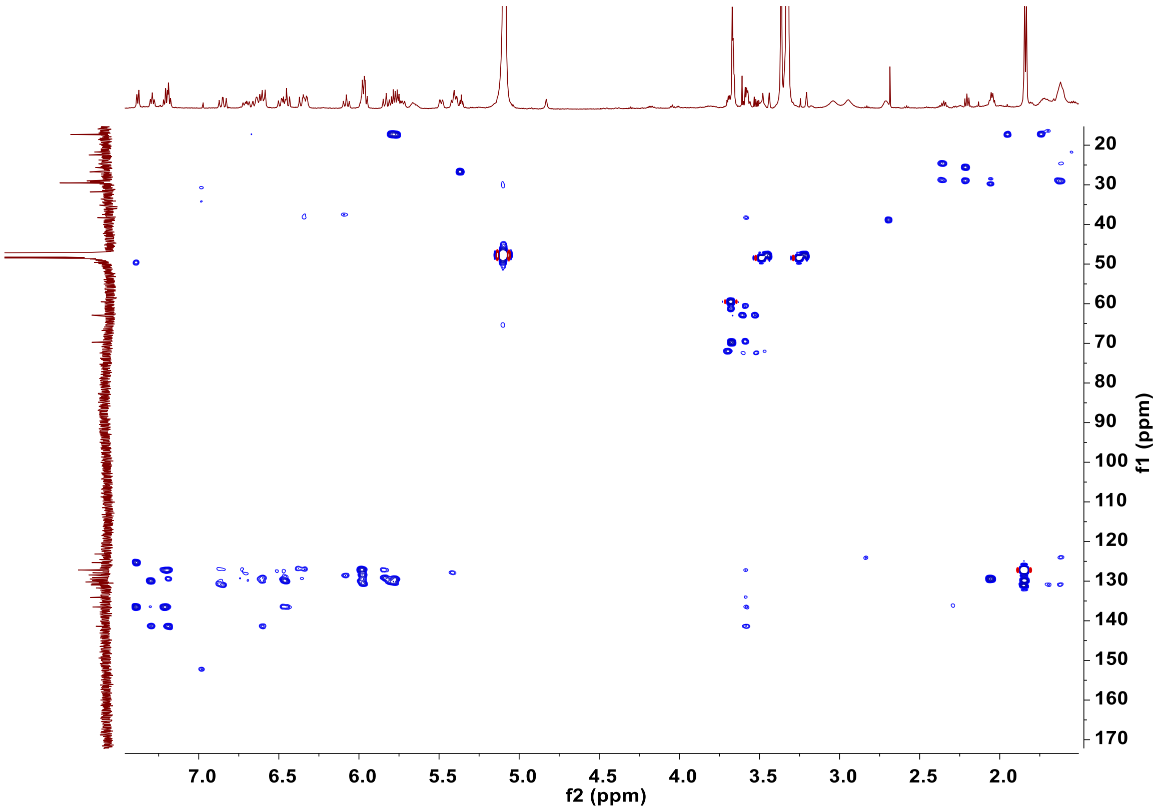

Figure S24. HMBC spectrum of compound **2** in CD_3OD (600 MHz)

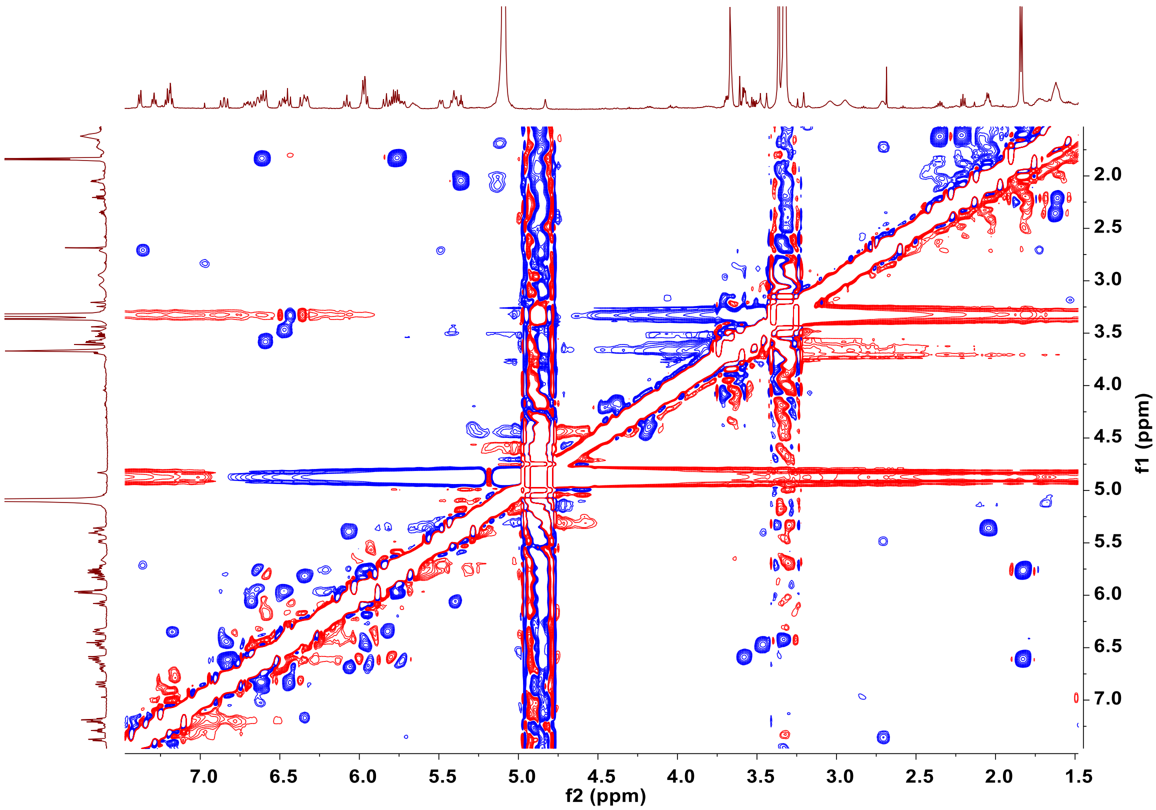

Figure S25. NOESY spectrum of compound **2** in CD_3OD (600 MHz)

Table S1. ^{1}H and ^{13}C NMR data of **1** in DMSO-d_6 (600 MHz)[a]

	**1**	
Position	δ_C, type	δ_H (J in Hz)
1	[b]	-
2	39.8, CH_2	2.60, m<br>2.47, m
3	42.1, CH	3.49, m
4	142.8, C	-
5	127.7, CH	7.28, d (7.6)
6	127.5, CH	7.23, t (7.0)
7	126.0, CH	7.19, t (7.6)
8	130.2, CH	7.12, d (7.5)
9	137.1, C	-
10	130.9, CH	6.80, d (10.6)
11	130.9, CH	6.42, m
12	129.5, CH	6.38, m
13	130.7, CH	6.88, t (13.0)
14	127.6, CH	5.87, t (11.3)
15	130.6, CH	5.98, m
16	128.0, CH	6.64, m
17	131.6, CH	5.79, m
18	18.6, CH_3	1.81, d (5.28)
1'	174.7, C	-
2'	42.1, CH_2	2.80, m
3'	131.8, CH	5.68, m
4'	125.9, CH	6.32, m
5'	126.0, CH	5.60, d (11.4)
6'	[b]	-
7'	124.3, CH	6.54, d (9.7)
8'	132.3 CH	5.73, m
9'	38.0, CH	2.63, m
10'	32.9 CH_2	1.51, m<br>1.40, m
11'	37.5 CH	3.12, m
12'	134.6 CH	5.24, m
13'	128.5, CH	6.00, m
14'	128.7, CH	6.32, m
15'	129.2 CH	6.72, dd (14.6, 10.7)
16'	127.9, CH	5.98, m
17'	130.6, CH	5.98, m
18'	127.2, CH	6.64, m
19'	131.6, CH	5.79, m
20'	18.6, CH_3	1.80, d (5.28)

[a] the ^{13}C chemical shifts were obtained by combination of HSQC and HMBC analysis

[b] not detected

Table S2. Antibacterial activity of compounds **1** and **2** against the multi-drug resistant *Enterococcus faecalis* CCARM 5172

Strain	MIC (μM)			
	**1**	**2**	Ciprofloxacin	Tetracycline
Enterococcus faecalis CCARM 5172	22.2	22.2	17.0	>112.5

Table S3. Bacteria and plasmids used in this study

Strains or plasmids	Description	Reference or source
S. youssoufiensis OUC6819	wild type, isolated from the reeds rhizosphere soil	[1, 2]
ΔdtlA	*dtlA* inactivation mutant of *S. youssoufiensis* OUC6819	[3]

Table S4. The computational method and coordinates of (3*R*,9'*R*,11'*S*)-**1** for ECD calculation

```
td(nstates=50) cam-b3lyp/6-31g(d) scrf=(solvent=methanol)
```

C	2.93553	1.57193	-1.56795
C	2.00222	1.51061	-2.59321
C	0.7781	2.14802	-2.43943
C	0.45205	2.84476	-1.27053
C	1.38924	2.88771	-0.21354
C	2.62315	2.25099	-0.39395
C	1.05815	3.57952	1.10421
C	0.46546	2.62776	2.19816
C	2.22959	4.39434	1.69627
C	-0.93223	2.20548	1.85859
C	-1.3238	0.94016	1.67836
C	-0.83734	3.57148	-1.2155
C	1.31405	1.41581	2.61658
C	1.06835	0.12132	1.81147
C	-0.41832	-0.20506	1.70986
C	1.87081	-0.99014	2.42998
C	-0.83995	-1.47881	1.5619
C	-2.20635	-1.92088	1.33549
C	-2.05375	3.04607	-1.43789
C	2.79842	-1.75929	1.84194
C	-2.36279	1.6409	-1.62516
C	-3.61288	1.17404	-1.80741
C	-3.92197	-0.23637	-1.93315
C	-5.14346	-0.7818	-2.1075
C	-6.41083	-0.07772	-2.21457
C	-7.58089	-0.70357	-2.39115
C	-8.90844	-0.02626	-2.50884
C	3.21229	-1.72455	0.44946
C	4.16168	-2.53315	-0.05735
C	4.57251	-2.50755	-1.44724
C	5.51275	-3.28249	-2.02459
C	6.31675	-4.30959	-1.38213
C	7.23162	-5.03714	-2.03441
C	8.08047	-6.1028	-1.4185
C	-2.54707	-3.20391	1.14103
C	-3.95294	-3.66642	0.89343
C	2.60709	5.64246	0.92097
O	2.72526	6.73011	1.43397
O	2.83225	5.50992	-0.39724
C	-4.60931	-4.34269	2.09103
O	-5.49687	-5.15606	1.99853
O	-4.17256	-3.95299	3.29807
H	3.90488	1.09547	-1.67262
H	2.23036	0.98809	-3.51654
H	0.05949	2.13567	-3.25224

H	3.36124	2.26764	0.40138
H	0.25662	4.29807	0.90582
H	0.38004	3.27345	3.08242
H	3.13061	3.78202	1.81048
H	1.9605	4.74055	2.69515
H	-1.67	2.99971	1.77839
H	-2.37162	0.75514	1.46925
H	-0.77809	4.63773	-1.00678
H	2.38199	1.65379	2.58334
H	1.08614	1.20323	3.66774
H	1.41204	0.29336	0.78735
H	1.66725	-1.16102	3.48652
H	-0.09237	-2.26826	1.5831
H	-2.99456	-1.17276	1.29145
H	-2.90677	3.72237	-1.42398
H	3.30265	-2.50276	2.45814
H	-1.53901	0.93295	-1.57425
H	-4.43323	1.88746	-1.853
H	-3.06994	-0.91098	-1.87311
H	-5.20507	-1.86628	-2.18222
H	-6.4044	1.00848	-2.14934
H	-7.58508	-1.79186	-2.45658
H	-8.81379	1.06061	-2.43241
H	-9.38731	-0.2627	-3.46659
H	-9.59531	-0.36714	-1.72492
H	2.72666	-1.01283	-0.21435
H	4.64593	-3.24429	0.60893
H	4.05729	-1.7832	-2.07572
H	5.70363	-3.1379	-3.08659
H	6.16481	-4.49348	-0.32035
H	7.383	-4.85215	-3.0982
H	7.92042	-7.06871	-1.91245
H	9.14649	-5.87005	-1.52808
H	7.86433	-6.22337	-0.3532
H	-1.77668	-3.97314	1.17253
H	-4.00839	-4.37677	0.06475
H	-4.59155	-2.81469	0.62815
H	2.70845	4.58479	-0.67988
H	-3.44394	-3.31328	3.18363

Standard orientation:

Center Number	Atomic Number	Atomic Type	Coordinates (Angstroms) X	Y	Z
1	6	0	2.935528	1.571925	-1.567954
2	6	0	2.002220	1.510612	-2.593205

3	6	0	0.778095	2.148017	-2.439430
4	6	0	0.452052	2.844758	-1.270528
5	6	0	1.389244	2.887708	-0.213544
6	6	0	2.623146	2.250990	-0.393949
7	6	0	1.058145	3.579523	1.104214
8	6	0	0.465461	2.627763	2.198160
9	6	0	2.229586	4.394342	1.696273
10	6	0	-0.932228	2.205476	1.858588
11	6	0	-1.323800	0.940161	1.678357
12	6	0	-0.837343	3.571477	-1.215497
13	6	0	1.314054	1.415812	2.616576
14	6	0	1.068345	0.121319	1.811469
15	6	0	-0.418318	-0.205057	1.709856
16	6	0	1.870806	-0.990142	2.429977
17	6	0	-0.839954	-1.478805	1.561903
18	6	0	-2.206350	-1.920879	1.335486
19	6	0	-2.053745	3.046074	-1.437891
20	6	0	2.798420	-1.759287	1.841941
21	6	0	-2.362788	1.640902	-1.625160
22	6	0	-3.612880	1.174037	-1.807414
23	6	0	-3.921971	-0.236374	-1.933153
24	6	0	-5.143457	-0.781798	-2.107503
25	6	0	-6.410832	-0.077721	-2.214568
26	6	0	-7.580891	-0.703567	-2.391146
27	6	0	-8.908444	-0.026257	-2.508841
28	6	0	3.212291	-1.724547	0.449462
29	6	0	4.161682	-2.533149	-0.057352
30	6	0	4.572514	-2.507549	-1.447238
31	6	0	5.512750	-3.282491	-2.024589
32	6	0	6.316747	-4.309585	-1.382134
33	6	0	7.231622	-5.037135	-2.034414
34	6	0	8.080474	-6.102801	-1.418503
35	6	0	-2.547074	-3.203911	1.141030
36	6	0	-3.952940	-3.666417	0.893428
37	6	0	2.607087	5.642464	0.920972
38	8	0	2.725260	6.730109	1.433966
39	8	0	2.832247	5.509918	-0.397242
40	6	0	-4.609306	-4.342694	2.091034
41	8	0	-5.496874	-5.156057	1.998531
42	8	0	-4.172560	-3.952987	3.298072
43	1	0	3.904879	1.095473	-1.672615
44	1	0	2.230362	0.988092	-3.516537
45	1	0	0.059492	2.135675	-3.252242
46	1	0	3.361236	2.267639	0.401376
47	1	0	0.256620	4.298066	0.905816
48	1	0	0.380037	3.273451	3.082422
49	1	0	3.130605	3.782015	1.810480
50	1	0	1.960502	4.740554	2.695153

51	1	0	−1.669996	2.999707	1.778392
52	1	0	−2.371619	0.755136	1.469251
53	1	0	−0.778085	4.637730	−1.006782
54	1	0	2.381987	1.653792	2.583336
55	1	0	1.086139	1.203225	3.667742
56	1	0	1.412039	0.293356	0.787349
57	1	0	1.667250	−1.161020	3.486515
58	1	0	−0.092368	−2.268262	1.583100
59	1	0	−2.994555	−1.172759	1.291451
60	1	0	−2.906766	3.722371	−1.423976
61	1	0	3.302649	−2.502756	2.458140
62	1	0	−1.539014	0.932947	−1.574247
63	1	0	−4.433232	1.887462	−1.852996
64	1	0	−3.069940	−0.910982	−1.873113
65	1	0	−5.205073	−1.866275	−2.182219
66	1	0	−6.404404	1.008480	−2.149335
67	1	0	−7.585075	−1.791861	−2.456579
68	1	0	−8.813788	1.060611	−2.432414
69	1	0	−9.387310	−0.262696	−3.466593
70	1	0	−9.595309	−0.367139	−1.724917
71	1	0	2.726661	−1.012833	−0.214354
72	1	0	4.645926	−3.244287	0.608928
73	1	0	4.057291	−1.783202	−2.075723
74	1	0	5.703626	−3.137902	−3.086588
75	1	0	6.164807	−4.493477	−0.320348
76	1	0	7.383004	−4.852145	−3.098202
77	1	0	7.920417	−7.068707	−1.912451
78	1	0	9.146493	−5.870052	−1.528081
79	1	0	7.864334	−6.223366	−0.353197
80	1	0	−1.776678	−3.973136	1.172525
81	1	0	−4.008386	−4.376768	0.064747
82	1	0	−4.591546	−2.814691	0.628152
83	1	0	2.708454	4.584787	−0.679881
84	1	0	−3.443937	−3.313281	3.183629

Table S5. The computational method and coordinates of (3*S*,9'*S*,11'*R*)-**1** for ECD calculation

td(nstates=50) cam-b3lyp/6-31g(d) scrf=(solvent=methanol)

C	-3.0321	-1.5602	1.46882
C	-2.10124	-1.50483	2.49828
C	-0.88152	-2.15094	2.34822
C	-0.55846	-2.84538	1.17624
C	-1.48919	-2.87354	0.11685
C	-2.72059	-2.23853	0.29555
C	-1.16116	-3.56533	-1.19788
C	-0.54763	-2.638	-2.29843
C	-2.35848	-4.34471	-1.77158
C	0.87581	-2.28523	-1.98541
C	1.32718	-1.04228	-1.78927
C	0.71349	-3.60257	1.12705
C	-1.34371	-1.38148	-2.68721
C	-1.02793	-0.11513	-1.8623
C	0.47328	0.14257	-1.77809
C	-1.78496	1.04379	-2.44984
C	0.95424	1.39191	-1.60542
C	2.34245	1.77223	-1.39812
C	1.94267	-3.1168	1.36755
C	-2.68521	1.83096	-1.8432
C	2.29001	-1.72243	1.56815
C	3.54863	-1.2925	1.78002
C	3.89579	0.10792	1.91789
C	5.12692	0.61766	2.12769
C	6.36963	-0.12339	2.27027
C	7.55267	0.46658	2.48057
C	8.85548	-0.25051	2.63507
C	-3.10808	1.76869	-0.45444
C	-4.02514	2.59937	0.07569
C	-4.44401	2.54238	1.46234
C	-5.35314	3.33564	2.0641
C	-6.10964	4.41817	1.45574
C	-6.99588	5.1611	2.12979
C	-7.79578	6.28263	1.54831
C	2.73227	3.03193	-1.17127
C	4.16277	3.42872	-0.92065
C	-2.96947	-5.31076	-0.79028
O	-4.15142	-5.42409	-0.55762
O	-2.04263	-6.07752	-0.18898
C	4.72559	4.26751	-2.0542
O	5.58171	3.8953	-2.81765
O	4.19577	5.49749	-2.20565
H	-4.00041	-1.08094	1.57456
H	-2.32969	-0.98208	3.42178
H	-0.16663	-2.14933	3.16476

H	-3.45957	-2.26466	-0.49841
H	-0.38874	-4.31066	-0.99277
H	-0.51512	-3.27912	-3.1906
H	-3.15823	-3.69357	-2.12712
H	-2.01246	-4.92782	-2.63274
H	1.57818	-3.11351	-1.9339
H	2.38589	-0.90664	-1.59649
H	0.62301	-4.665	0.90861
H	-2.42092	-1.56995	-2.64077
H	-1.12031	-1.16137	-3.738
H	-1.36756	-0.29322	-0.83792
H	-1.5701	1.23779	-3.50023
H	0.24039	2.21252	-1.59013
H	3.10241	0.99503	-1.4065
H	2.7758	-3.81779	1.35959
H	-3.1569	2.61189	-2.4386
H	1.48767	-0.99119	1.50321
H	4.34684	-2.02956	1.84112
H	3.066	0.80719	1.83347
H	5.21812	1.69989	2.20446
H	6.33243	-1.20897	2.20383
H	7.58806	1.55427	2.54729
H	9.31429	-0.02897	3.60615
H	9.57426	0.06917	1.87105
H	8.73028	-1.33405	2.55502
H	-2.65858	1.01353	0.18629
H	-4.47536	3.35316	-0.56725
H	-3.96345	1.77422	2.06586
H	-5.55533	3.16157	3.11957
H	-5.94492	4.63209	0.40149
H	-7.16027	4.94597	3.18597
H	-7.59808	7.22318	2.07641
H	-8.87127	6.09145	1.64514
H	-7.56885	6.43107	0.48876
H	1.98506	3.82448	-1.15401
H	4.24041	4.00516	0.01064
H	4.79623	2.54588	-0.82446
H	-2.51281	-6.67388	0.42221
H	3.54945	5.692	-1.50774

Standard orientation:

Center Number	Atomic Number	Atomic Type	Coordinates (Angstroms) X	Y	Z
1	6	0	-3.032099	-1.560196	1.468821
2	6	0	-2.101239	-1.504832	2.498278

3	6	0	−0.881520	−2.150936	2.348215
4	6	0	−0.558463	−2.845379	1.176236
5	6	0	−1.489185	−2.873541	0.116848
6	6	0	−2.720593	−2.238528	0.295545
7	6	0	−1.161157	−3.565330	−1.197878
8	6	0	−0.547634	−2.638002	−2.298432
9	6	0	−2.358477	−4.344713	−1.771583
10	6	0	0.875808	−2.285229	−1.985406
11	6	0	1.327178	−1.042275	−1.789268
12	6	0	0.713486	−3.602569	1.127049
13	6	0	−1.343706	−1.381483	−2.687209
14	6	0	−1.027934	−0.115130	−1.862296
15	6	0	0.473282	0.142568	−1.778086
16	6	0	−1.784959	1.043792	−2.449841
17	6	0	0.954235	1.391910	−1.605423
18	6	0	2.342451	1.772230	−1.398117
19	6	0	1.942670	−3.116802	1.367545
20	6	0	−2.685212	1.830964	−1.843200
21	6	0	2.290014	−1.722430	1.568151
22	6	0	3.548634	−1.292498	1.780017
23	6	0	3.895787	0.107919	1.917890
24	6	0	5.126918	0.617659	2.127691
25	6	0	6.369627	−0.123389	2.270269
26	6	0	7.552668	0.466578	2.480574
27	6	0	8.855482	−0.250509	2.635072
28	6	0	−3.108079	1.768687	−0.454435
29	6	0	−4.025137	2.599369	0.075692
30	6	0	−4.444014	2.542377	1.462344
31	6	0	−5.353137	3.335638	2.064096
32	6	0	−6.109643	4.418165	1.455740
33	6	0	−6.995883	5.161104	2.129793
34	6	0	−7.795775	6.282631	1.548310
35	6	0	2.732269	3.031925	−1.171268
36	6	0	4.162774	3.428719	−0.920653
37	6	0	−2.969469	−5.310755	−0.790280
38	8	0	−4.151421	−5.424088	−0.557616
39	8	0	−2.042627	−6.077524	−0.188978
40	6	0	4.725590	4.267513	−2.054202
41	8	0	5.581709	3.895300	−2.817653
42	8	0	4.195768	5.497490	−2.205653
43	1	0	−4.000407	−1.080941	1.574559
44	1	0	−2.329687	−0.982081	3.421779
45	1	0	−0.166625	−2.149330	3.164759
46	1	0	−3.459569	−2.264655	−0.498406
47	1	0	−0.388742	−4.310661	−0.992768
48	1	0	−0.515118	−3.279120	−3.190603
49	1	0	−3.158232	−3.693569	−2.127118
50	1	0	−2.012457	−4.927815	−2.632739

51	1	0	1.578178	-3.113508	-1.933899
52	1	0	2.385889	-0.906638	-1.596491
53	1	0	0.623005	-4.664997	0.908608
54	1	0	-2.420916	-1.569951	-2.640772
55	1	0	-1.120313	-1.161372	-3.738001
56	1	0	-1.367557	-0.293215	-0.837915
57	1	0	-1.570101	1.237792	-3.500230
58	1	0	0.240388	2.212515	-1.590133
59	1	0	3.102407	0.995025	-1.406504
60	1	0	2.775795	-3.817792	1.359591
61	1	0	-3.156904	2.611890	-2.438595
62	1	0	1.487669	-0.991189	1.503211
63	1	0	4.346837	-2.029557	1.841119
64	1	0	3.065998	0.807194	1.833472
65	1	0	5.218124	1.699891	2.204456
66	1	0	6.332431	-1.208974	2.203834
67	1	0	7.588057	1.554269	2.547290
68	1	0	9.314288	-0.028966	3.606147
69	1	0	9.574260	0.069173	1.871047
70	1	0	8.730280	-1.334049	2.555021
71	1	0	-2.658580	1.013534	0.186291
72	1	0	-4.475355	3.353163	-0.567247
73	1	0	-3.963447	1.774220	2.065858
74	1	0	-5.555327	3.161566	3.119570
75	1	0	-5.944922	4.632091	0.401491
76	1	0	-7.160272	4.945971	3.185968
77	1	0	-7.598078	7.223175	2.076413
78	1	0	-8.871266	6.091447	1.645138
79	1	0	-7.568854	6.431066	0.488757
80	1	0	1.985056	3.824484	-1.154007
81	1	0	4.240410	4.005158	0.010641
82	1	0	4.796234	2.545876	-0.824458
83	1	0	-2.512814	-6.673875	0.422207
84	1	0	3.549449	5.692004	-1.507741

Table S6. The computational method and coordinates of (5*R*,7'*R*,9'*S*)-**2** for ECD calculation

td(nstates=50) cam-b3lyp/6-31g(d) scrf=(solvent=methanol)

C	-1.48051	-2.38644	3.70146
C	-2.8198	-2.0489	3.73695
C	-3.5159	-1.92951	2.55287
C	-2.90035	-2.12779	1.3187
C	-1.53942	-2.45832	1.27923
C	-0.86028	-2.59127	2.48584
C	-0.81411	-2.70012	-0.04532
C	0.09824	-1.53886	-0.56492
C	-0.05002	-4.00612	-0.02377
C	-0.70716	-0.34264	-0.98953
C	-0.50614	0.89893	-0.58272
C	-3.73842	-2.06458	0.08954
C	1.29699	-1.11319	0.29916
C	1.02272	0.08684	1.22943
C	0.49124	1.2764	0.43645
C	2.197	0.3386	2.1576
C	0.82239	2.53113	0.70374
C	0.30097	3.78423	0.03868
C	-4.53243	-1.07393	-0.29553
C	3.43837	0.71018	1.8756
C	-4.69089	0.23141	0.34209
C	-5.55015	1.14834	-0.09273
C	-5.70033	2.45982	0.53134
C	-6.53343	3.43082	0.16313
C	-7.48674	3.41081	-0.94744
C	-8.27706	4.43317	-1.23505
C	-9.26927	4.46769	-2.35763
C	4.00097	1.05426	0.56988
C	5.2772	1.386	0.39922
C	5.84397	1.73404	-0.90085
C	7.10459	2.07489	-1.15967
C	8.21014	2.18148	-0.20613
C	9.43768	2.52498	-0.56345
C	10.60472	2.64917	0.3685
C	-0.33491	-5.03223	-0.79731
C	0.42278	-6.3358	-0.77061
C	1.10438	-6.63749	-2.08186
O	2.2702	-6.8246	-2.21519
O	0.25975	-6.68665	-3.09862
C	1.41663	4.47808	-0.70493
O	2.09025	5.35036	-0.25944
O	1.59651	3.99898	-1.92443
H	-0.92153	-2.49697	4.61221
H	-3.32118	-1.89586	4.67459
H	-4.56436	-1.70049	2.57941

H	0.17826	-2.85886	2.48607
H	-1.56814	-2.80803	-0.8101
H	0.51183	-1.95002	-1.48181
H	0.77537	-4.09103	0.6624
H	-1.48034	-0.52513	-1.71563
H	-1.11667	1.6739	-1.00463
H	-3.71509	-2.9395	-0.53645
H	1.65887	-1.94042	0.89466
H	2.10734	-0.85371	-0.37034
H	0.19874	-0.19922	1.87045
H	1.98894	0.13989	3.1952
H	1.54226	2.71669	1.47859
H	-0.50332	3.58795	-0.6512
H	-0.05021	4.47365	0.79485
H	-5.11024	-1.22402	-1.19213
H	4.12806	0.78154	2.70068
H	-4.0689	0.45372	1.19057
H	-6.16715	0.91758	-0.94319
H	-5.06249	2.64514	1.3789
H	-6.51583	4.34159	0.73777
H	-7.5431	2.52316	-1.55294
H	-8.2197	5.32279	-0.62707
H	-9.04711	5.28357	-3.03896
H	-9.2668	3.54145	-2.91919
H	-10.272	4.63604	-1.97631
H	3.34651	1.0467	-0.28206
H	5.9273	1.39946	1.2562
H	5.15551	1.70608	-1.72844
H	7.35591	2.30095	-2.18233
H	8.00989	1.96996	0.82968
H	9.63743	2.73699	-1.60247
H	11.01103	3.65583	0.34027
H	10.32415	2.42108	1.38956
H	11.40345	1.97553	0.07272
H	-1.15549	-4.97432	-1.49205
H	1.17967	-6.33207	-0.00134
H	-0.26259	-7.15503	-0.5744
H	0.73009	-6.87745	-3.90025
H	2.32577	4.44165	-2.33987

Standard orientation:

Center Number	Atomic Number	Atomic Type	Coordinates (Angstroms) X	Y	Z
1	6	0	1.480510	2.386438	3.701459
2	6	0	2.819800	2.048896	3.736954

3	6	0	3.515901	1.929512	2.552874
4	6	0	2.900345	2.127786	1.318701
5	6	0	1.539420	2.458323	1.279232
6	6	0	0.860282	2.591265	2.485844
7	6	0	0.814110	2.700115	-0.045323
8	6	0	-0.098236	1.538855	-0.564921
9	6	0	0.050018	4.006116	-0.023768
10	6	0	0.707155	0.342636	-0.989529
11	6	0	0.506143	-0.898930	-0.582722
12	6	0	3.738418	2.064584	0.089540
13	6	0	-1.296992	1.113188	0.299164
14	6	0	-1.022723	-0.086843	1.229431
15	6	0	-0.491238	-1.276398	0.436451
16	6	0	-2.197004	-0.338596	2.157604
17	6	0	-0.822390	-2.531125	0.703737
18	6	0	-0.300970	-3.784225	0.038682
19	6	0	4.532427	1.073926	-0.295527
20	6	0	-3.438370	-0.710175	1.875601
21	6	0	4.690888	-0.231405	0.342091
22	6	0	5.550149	-1.148343	-0.092730
23	6	0	5.700333	-2.459815	0.531344
24	6	0	6.533428	-3.430819	0.163134
25	6	0	7.486739	-3.410807	-0.947441
26	6	0	8.277059	-4.433171	-1.235054
27	6	0	9.269268	-4.467690	-2.357626
28	6	0	-4.000971	-1.054257	0.569878
29	6	0	-5.277200	-1.385998	0.399215
30	6	0	-5.843973	-1.734040	-0.900845
31	6	0	-7.104594	-2.074890	-1.159673
32	6	0	-8.210144	-2.181481	-0.206133
33	6	0	-9.437679	-2.524981	-0.563454
34	6	0	-10.604723	-2.649174	0.368502
35	6	0	0.334905	5.032233	-0.797312
36	6	0	-0.422783	6.335804	-0.770614
37	6	0	-1.104380	6.637490	-2.081856
38	8	0	-2.270196	6.824595	-2.215192
39	8	0	-0.259746	6.686646	-3.098615
40	6	0	-1.416629	-4.478081	-0.704932
41	8	0	-2.090254	-5.350356	-0.259444
42	8	0	-1.596510	-3.998978	-1.924434
43	1	0	0.921525	2.496973	4.612205
44	1	0	3.321180	1.895864	4.674586
45	1	0	4.564363	1.700488	2.579407
46	1	0	-0.178264	2.858863	2.486070
47	1	0	1.568140	2.808029	-0.810100
48	1	0	-0.511832	1.950023	-1.481805
49	1	0	-0.775370	4.091033	0.662396
50	1	0	1.480337	0.525125	-1.715632

51	1	0	1.116673	−1.673897	−1.004626
52	1	0	3.715090	2.939496	−0.536450
53	1	0	−1.658866	1.940420	0.894655
54	1	0	−2.107337	0.853705	−0.370337
55	1	0	−0.198740	0.199217	1.870445
56	1	0	−1.988941	−0.139894	3.195195
57	1	0	−1.542262	−2.716689	1.478592
58	1	0	0.503315	−3.587953	−0.651200
59	1	0	0.050209	−4.473654	0.794845
60	1	0	5.110244	1.224024	−1.192125
61	1	0	−4.128057	−0.781537	2.700678
62	1	0	4.068896	−0.453724	1.190572
63	1	0	6.167152	−0.917580	−0.943189
64	1	0	5.062491	−2.645141	1.378901
65	1	0	6.515827	−4.341593	0.737767
66	1	0	7.543104	−2.523163	−1.552935
67	1	0	8.219701	−5.322791	−0.627070
68	1	0	9.047113	−5.283569	−3.038957
69	1	0	9.266805	−3.541445	−2.919188
70	1	0	10.272001	−4.636044	−1.976306
71	1	0	−3.346509	−1.046702	−0.282057
72	1	0	−5.927295	−1.399464	1.256198
73	1	0	−5.155514	−1.706080	−1.728443
74	1	0	−7.355907	−2.300952	−2.182327
75	1	0	−8.009894	−1.969962	0.829683
76	1	0	−9.637433	−2.736992	−1.602473
77	1	0	−11.011033	−3.655829	0.340271
78	1	0	−10.324147	−2.421076	1.389555
79	1	0	−11.403450	−1.975533	0.072721
80	1	0	1.155491	4.974324	−1.492046
81	1	0	−1.179670	6.332072	−0.001337
82	1	0	0.262589	7.155028	−0.574403
83	1	0	−0.730094	6.877446	−3.900246
84	1	0	−2.325768	−4.441653	−2.339866

Table S7. The computational method and coordinates of (5*S*,7'*S*,9'*R*)-**2** for ECD calculation

td(nstates=50) cam-b3lyp/6-31g(d) scrf=(solvent=methanol)

C	-3.12131	-3.94306	2.46963
C	-1.98228	-4.67227	2.18589
C	-1.13148	-4.23605	1.19245
C	-1.38538	-3.07117	0.47117
C	-2.53988	-2.33371	0.75572
C	-3.38961	-2.79239	1.7564
C	-2.90797	-1.06888	-0.02544
C	-3.14821	0.15166	0.91252
C	-4.10287	-1.37581	-0.90341
C	-3.76625	1.31851	0.18816
C	-3.18561	2.49307	0.01201
C	-0.45425	-2.68473	-0.62284
C	-1.87323	0.59739	1.64066
C	-0.99618	1.55114	0.80844
C	-1.80612	2.79073	0.44152
C	0.27848	1.84239	1.55672
C	-1.29231	4.01454	0.46607
C	-1.97434	5.30489	0.09483
C	0.8661	-2.56609	-0.54903
C	1.51599	1.84463	1.08381
C	1.70195	-2.69569	0.64304
C	3.03005	-2.64896	0.60387
C	3.86528	-2.76453	1.79591
C	5.19548	-2.74469	1.84715
C	6.12664	-2.60356	0.72668
C	7.44155	-2.60455	0.88062
C	8.44057	-2.46614	-0.2278
C	1.9318	1.61724	-0.30009
C	3.19813	1.69227	-0.69724
C	3.61441	1.47906	-2.08071
C	4.85158	1.55748	-2.56606
C	6.07638	1.87508	-1.82992
C	7.26641	1.93845	-2.40644
C	8.5498	2.26127	-1.70309
C	-4.06619	-1.39292	-2.21848
C	-5.19382	-1.75948	-3.15696
C	-6.54737	-1.89416	-2.49764
O	-7.09809	-2.92793	-2.318
O	-7.13557	-0.76935	-2.11059
C	-2.15342	6.26842	1.25481
O	-2.10647	7.44772	1.13399
O	-2.41654	5.74153	2.43972
H	-3.80111	-4.26931	3.2348
H	-1.76332	-5.5759	2.72384
H	-0.26009	-4.81578	0.95438

H	-4.28529	-2.24735	1.98716
H	-2.08598	-0.81395	-0.67666
H	-3.8694	-0.15185	1.66285
H	-5.01576	-1.61843	-0.38253
H	-4.76938	1.18836	-0.17499
H	-3.7431	3.26638	-0.48056
H	-0.90557	-2.50906	-1.5833
H	-1.28668	-0.26412	1.93236
H	-2.15593	1.10872	2.55599
H	-0.75812	1.05669	-0.12603
H	0.15005	2.0634	2.60402
H	-0.26834	4.12865	0.77143
H	-1.40127	5.83467	-0.6537
H	-2.96534	5.1517	-0.31149
H	1.3917	-2.32705	-1.45829
H	2.31462	2.0571	1.77519
H	1.20885	-2.82143	1.59014
H	3.51661	-2.52184	-0.34701
H	3.3326	-2.87971	2.72462
H	5.65415	-2.84446	2.81655
H	5.71836	-2.49552	-0.26289
H	7.84932	-2.71302	1.87375
H	7.95486	-2.36133	-1.19024
H	9.07337	-1.59873	-0.06571
H	9.09305	-3.33343	-0.2643
H	1.17224	1.39231	-1.02775
H	3.9558	1.92247	0.03078
H	2.82345	1.2363	-2.77007
H	4.98274	1.37123	-3.61877
H	5.99918	2.06614	-0.77387
H	7.34275	1.74729	-3.46571
H	9.00945	3.14592	-2.13364
H	8.39054	2.43737	-0.64624
H	9.26095	1.44805	-1.81285
H	-3.14666	-1.15389	-2.72544
H	-5.26506	-1.00999	-3.93888
H	-4.97545	-2.70279	-3.63879
H	-6.62145	-0.00014	-2.30616
H	-2.393	4.79451	2.41952

Standard orientation:

Center Number	Atomic Number	Atomic Type	Coordinates (Angstroms) X	Y	Z
1	6	0	3.121311	-3.943062	-2.469626
2	6	0	1.982280	-4.672265	-2.185891

3	6	0	1.131483	-4.236052	-1.192451
4	6	0	1.385379	-3.071174	-0.471168
5	6	0	2.539879	-2.333712	-0.755718
6	6	0	3.389605	-2.792391	-1.756403
7	6	0	2.907966	-1.068882	0.025444
8	6	0	3.148212	0.151658	-0.912519
9	6	0	4.102871	-1.375814	0.903406
10	6	0	3.766254	1.318513	-0.188161
11	6	0	3.185609	2.493070	-0.012008
12	6	0	0.454248	-2.684730	0.622843
13	6	0	1.873230	0.597386	-1.640664
14	6	0	0.996183	1.551135	-0.808438
15	6	0	1.806123	2.790729	-0.441521
16	6	0	-0.278481	1.842391	-1.556715
17	6	0	1.292307	4.014535	-0.466070
18	6	0	1.974337	5.304891	-0.094825
19	6	0	-0.866099	-2.566091	0.549034
20	6	0	-1.515991	1.844629	-1.083812
21	6	0	-1.701946	-2.695692	-0.643039
22	6	0	-3.030050	-2.648964	-0.603872
23	6	0	-3.865276	-2.764526	-1.795907
24	6	0	-5.195480	-2.744690	-1.847147
25	6	0	-6.126643	-2.603561	-0.726683
26	6	0	-7.441550	-2.604552	-0.880615
27	6	0	-8.440572	-2.466139	0.227802
28	6	0	-1.931800	1.617244	0.300093
29	6	0	-3.198126	1.692266	0.697243
30	6	0	-3.614409	1.479059	2.080711
31	6	0	-4.851580	1.557475	2.566057
32	6	0	-6.076378	1.875076	1.829915
33	6	0	-7.266412	1.938448	2.406436
34	6	0	-8.549801	2.261267	1.703085
35	6	0	4.066192	-1.392915	2.218482
36	6	0	5.193824	-1.759478	3.156956
37	6	0	6.547372	-1.894155	2.497638
38	8	0	7.098091	-2.927928	2.318000
39	8	0	7.135569	-0.769349	2.110591
40	6	0	2.153425	6.268418	-1.254814
41	8	0	2.106465	7.447718	-1.133990
42	8	0	2.416544	5.741527	-2.439719
43	1	0	3.801108	-4.269307	-3.234803
44	1	0	1.763315	-5.575900	-2.723842
45	1	0	0.260087	-4.815784	-0.954383
46	1	0	4.285294	-2.247346	-1.987157
47	1	0	2.085985	-0.813950	0.676658
48	1	0	3.869400	-0.151853	-1.662850
49	1	0	5.015760	-1.618431	0.382534
50	1	0	4.769375	1.188356	0.174992

51	1	0	3. 743095	3. 266380	0. 480562
52	1	0	0. 905574	−2. 509056	1. 583301
53	1	0	1. 286680	−0. 264116	−1. 932356
54	1	0	2. 155929	1. 108725	−2. 555994
55	1	0	0. 758123	1. 056689	0. 126032
56	1	0	−0. 150046	2. 063402	−2. 604021
57	1	0	0. 268341	4. 128650	−0. 771429
58	1	0	1. 401274	5. 834670	0. 653698
59	1	0	2. 965340	5. 151695	0. 311490
60	1	0	−1. 391697	−2. 327050	1. 458285
61	1	0	−2. 314623	2. 057104	−1. 775193
62	1	0	−1. 208852	−2. 821433	−1. 590136
63	1	0	−3. 516614	−2. 521839	0. 347010
64	1	0	−3. 332596	−2. 879705	−2. 724621
65	1	0	−5. 654151	−2. 844464	−2. 816552
66	1	0	−5. 718356	−2. 495515	0. 262890
67	1	0	−7. 849319	−2. 713022	−1. 873754
68	1	0	−7. 954861	−2. 361325	1. 190245
69	1	0	−9. 073365	−1. 598725	0. 065710
70	1	0	−9. 093051	−3. 333431	0. 264301
71	1	0	−1. 172238	1. 392311	1. 027749
72	1	0	−3. 955802	1. 922473	−0. 030784
73	1	0	−2. 823452	1. 236295	2. 770073
74	1	0	−4. 982740	1. 371229	3. 618767
75	1	0	−5. 999178	2. 066137	0. 773865
76	1	0	−7. 342750	1. 747285	3. 465709
77	1	0	−9. 009453	3. 145920	2. 133636
78	1	0	−8. 390539	2. 437369	0. 646236
79	1	0	−9. 260951	1. 448050	1. 812852
80	1	0	3. 146658	−1. 153894	2. 725441
81	1	0	5. 265058	−1. 009990	3. 938882
82	1	0	4. 975449	−2. 702788	3. 638793
83	1	0	6. 621451	−0. 000136	2. 306157
84	1	0	2. 392999	4. 794505	−2. 419520

References

1. Che, Q.; Zhu, T.; Qi, X.; Mándi, A.; Kurtán, T.; Mo, X.; Li, D. Hybrid Isoprenoids from a Reeds Rhizosphere Soil Derived Actinomycete *Streptomyces* sp. CHQ-64. *Org. Lett.* **2012**, *14,* 3438-3441.

2. Yao, T.; Liu, J.; Liu, Z.; Li, T.; Li, H.; Che, Q.; Zhu, T.; Li, D.; Li, W. Genome mining of cyclodipeptide synthases unravels unusual tRNA-dependent diketopiperazine-terpene biosynthetic machinery. *Nat. Commun.* **2018**, *9*, 4091

3. Li, H.; Liu, J.; Deng, Z.; Li, T.; Liu, Z.; Che, Q.; Li, W. Genetic Manipulation of an Aminotransferase Family Gene *dtlA* Activates Youssoufenes in Marine-Derived *Streptomyces youssoufiensis*. *Org Lett*. **2020**, *17*, 729-733.
